# A Day in the Life: Characterization of Doctoral Bench Research in Synthetic Chemistry Using Phenomenological Case Studies

**DOI:** 10.1021/acs.jchemed.2c00809

**Published:** 2022-12-28

**Authors:** Elizabeth W. Kelley

**Affiliations:** Chemistry Department, University of Chicago, 5735 S Ellis Ave, Chicago, Illinois 60637, United States

**Keywords:** Chemical Education Research, First-Year Undergraduate/General, Second-Year Undergraduate, Upper-Division Undergraduate, Graduate Education/Research, Curriculum, Laboratory Instruction, Organic Chemistry, Hands-On Learning/Manipulatives, Instrumental Methods, Laboratory Equipment/Apparatus, Laboratory Management, Standards National/State

## Abstract

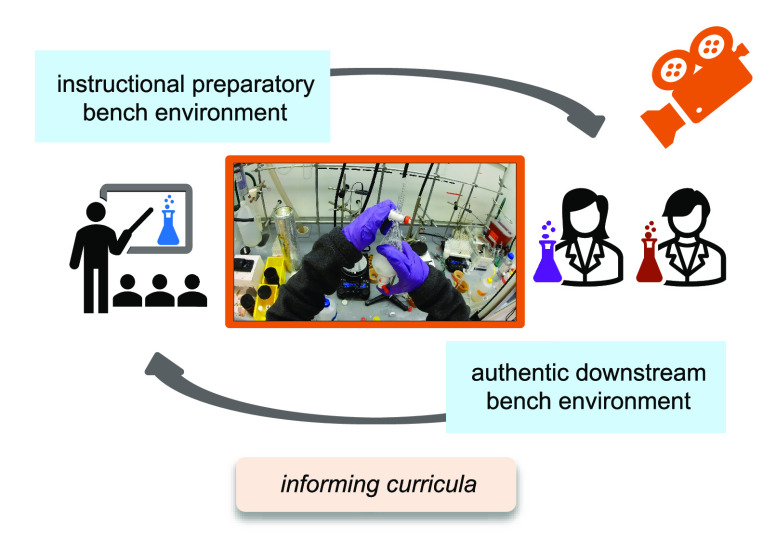

Despite decades of reform efforts, STEM education continues to face calls for improvement, especially regarding the teaching laboratory. Establishing an empirical understanding of the types of hands-on, psychomotor skills that students need to learn to succeed in downstream careers could help ensure laboratory courses are promoting authentic learning. Therefore, this paper reports phenomenological grounded theory case studies characterizing the nature of benchwork in synthetic organic chemistry graduate research. Through first-person video data and retrospective interviews, the results illustrate how organic chemistry students use psychomotor skills to conduct doctoral research and where they acquired those skills. By understanding the role that psychomotor skills play in authentic benchwork and the role that teaching laboratories play in the development of those skills, chemical educators could revolutionize undergraduate laboratory experiences by enabling evidence-based incorporation of the psychomotor component into laboratory learning objectives.

The purpose, role, and effectiveness of laboratory education are currently subjects of renewed debate.^1−8^ Evidence is building that current practices in undergraduate laboratory courses may not promote desired learning outcomes as effectively as hoped. As a result, curriculum reform efforts in the laboratory sciences are increasingly popular, such as replacing oft-disparaged replicative cookbook experiments with inquiry-based experiments and course-based undergraduate research experiences (CUREs).^9−14^ Concurrently, doubts have also been raised as to whether hands-on laboratories themselves are a worthwhile investment or as pedagogically unique as previously assumed.^15−30^ In the wake of widespread precedents for virtual learning set by the COVID-19 pandemic, some educators are exploring the possibilities afforded by the adoption of increasingly sophisticated virtual laboratories whereas others eagerly return to classrooms with renewed conviction in the value of hands-on laboratories. As a result of the enthusiasm for this topic, publications about the laboratory are proliferating in discipline-based education research (DBER) journals across STEM fields.^3,15,31,32^

Regardless of the ultimate design elements, content, or modality that future curricula adopt, laboratory education is clearly on the cusp of a revolution. However, while the DBER community’s understanding of the landscape of effective laboratory practices is accelerating, the frontier is far from conquered. Many unknowns persist. One glaring black hole compromising educators’ abilities to effectively design what are here dubbed as next-generation laboratory standards (NGLSs) is the lack of a shared, empirical understanding for what the product should look like: what do students need to know and be able to do to be successful bench scientists downstream, after they complete their formal education? While many students in chemistry courses do not ultimately become chemists, one rationale for offering such experiences is to prepare students for laboratory careers. Prior research has revealed important aspects of affective and cognitive domains to cultivate in STEM education as well as general traits that support good scientific practices.^33−36^ However, while there have been prior research efforts regarding the cultivation of psychomotor skills (e.g., developing rubrics to assess execution of specific experimental techniques),^37−40^ there is not yet a literature basis for characterizing the specific types of psychomotor skills that students need to learn for future chemistry careers. Likewise, the “neglected materiality” component of chemistry teaching laboratories has recently been highlighted,^41^ suggesting that chemistry education research has focused less on the physical domain of laboratory science experiences than the nonphysical in recent years. Since laboratories are fundamentally physical environments requiring practitioner expertise with a variety of specialized tools and techniques, further deliberate investigation into the development of chemists’ psychomotor skills is warranted.

A systematic characterization of requisite psychomotor skills would therefore assist curriculum reform efforts to develop NGLSs for psychomotor skills relevant to laboratory-based careers. While many chemistry laboratory courses include classical techniques shared in common across institutions and decades (e.g., gas chromatography, distillation), each course designer exercises agency over the experiments students will conduct. With this agency comes diversity in the tools and techniques which students are exposed to, trained in, and assessed on, based on the course designer’s prior experiences, personal skills, and beliefs about the importance of various skills. Additionally, it is also important to recognize that most existing evidence about laboratory learning (and learning in general) revolves around introductory undergraduate students.^3,35^ The dearth of attention to professional chemists adds uncertainty to the development of NGLSs without knowledge of how laboratory learning sets a foundation for downstream careers. For example, do graduate students rely on cookbook techniques they learned in traditional undergraduate laboratories, and are those techniques effectively fostered in inquiry classrooms? What types of skills do industrial bench chemists need mastered to meet employer expectations, and does that differ from what academic chemists learn? How do introductory students later build on foundational experimental skills to master complex techniques? Although “the bench” is not the only destination for chemistry students, the bench is nonetheless an integral part of experimental science in downstream careers.

Therefore, to maximize the likelihood that future laboratory course reform endeavors will lead to substantive improvements in the competencies of the STEM workforce, there is a pressing need for evidence regarding what laboratory skills students need for downstream career success and how to most effectively build those skills. Empirically elucidating the nature of the skills that laboratory-based chemists use could generate a shared, empirical understanding of NGLSs that supports educators in their quest to improve STEM education. This paper therefore reports on the validation and implementation of a method used to characterize psychomotor skills executed by practicing chemists (here, PhD chemistry student workers) in an authentic laboratory workplace environment. Understanding the skills practicing chemists use could then inform subsequent undergraduate laboratory reforms.

## Research Questions

This study sought to characterize the nature of chemical benchwork in an authentic laboratory workplace by asking two driving questions:

- How do chemists use their psychomotor skills when conducting bench research?
- Where did those chemists learn the psychomotor skills?

## Frameworks

Due to the dearth of information about professional chemists’ experiences in laboratory workplaces, a phenomenological grounded theory case study framework with naturalistic and retrospective data was adopted to capture a set of participating chemists’ first-person experiences, thus fueling an emergent understanding of such environments.^42^ Phenomenology is the study of an individual’s experience of phenomena through their own perspective, enabling the researcher to accept their reports as true interpretations of their experiences.^43,44^ Phenomenology is distinct from many other frameworks in that it focuses around the individual’s experience of phenomena rather than the researchers’ outsider perceptions of the experience. Phenomenology also often focuses on illuminating the individual’s intentionality in engaging with the world in conjunction with understanding their lived experiences. This phenomenology was pursued using a highly authentic naturalistic observation protocol coupled with retrospective interviews which involved the participants in contextualizing and validating the observations.^45,46^ Due to the inherently physical nature of psychomotor skills, participants were outfitted with action cameras on their heads to capture their first-person perspectives as organically and accurately as possible; the phenomenology was almost literally “through their eyes”. Through retrospective interviews, the participants later provided contextualization of those observed experiences.

These naturalistic observations and participant perspectives were analyzed via grounded theory in tandem with data collection. Grounded theory is a methodological framework that relies on emerging themes to concurrently guide data collection and analysis, enabling the researcher to begin a study with minimal preconceived assumptions about the prospective data.^47−49^ Following where the data leads then results in a tailored framework based authentically on the data. Grounded theory was pursued using case studies to enable a deep dive into the psychomotor skills used by individual chemists and the origins of those skills for each individual.

**Figure 1:**
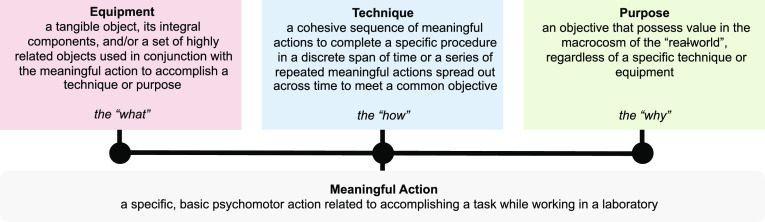
Phenomenological grounded theory led to the development of the MA–ETP framework to characteristic psychomotor actions in a graduate synthetic chemistry research laboratory. Psychomotor actions were coded with inductive open basic codes (Meaningful Actions) which were later assigned one each of an Equipment, Technique, and Purpose code during axial coding.

Together, these frameworks and reflective memoing during the study led to the development of a novel, emergent framework (here dubbed the MA–ETP framework, Figure 1) through which the final data was analyzed. Inductive open basic coding of the psychomotor actions that participants engaged in during naturalistic observation yielded descriptions of those actions in terms of both the physical action itself and the underlying rationale for engaging in it. These “meaningful actions” were connected to each other in terms of the equipment used, techniques they were a part of, and the real-world purposes that they were meant to achieve (ETP). The MA–ETP framework formed the backbone of the data analysis.

## Methodology

Purposeful convenience sampling led to the recruitment of two synthetic chemistry PhD candidates and their research group in an academic laboratory.^50^ Over the course of a few months with 6 recording sessions and 8 interviews, the study activities led to in-depth coding that yielded rich data about the role of psychomotor actions at “the bench”.

### Recruitment with Purposeful Convenience Sampling

Academic research laboratories were targeted as sources of potential participants by convenience sampling. Purposeful sampling was then utilized to specifically target wet synthetic chemists due to (1) the bench-intensive nature of synthetic chemistry research and (2) the author’s personal background in PhD-level synthetic chemistry (thus enabling accurate identification of psychomotor skills and materials used). Therefore, R1 Principal Investigators (PIs) with synthetic chemistry research laboratories in the United States were approached for consent to recruit their group members and conduct recording activities in the group’s laboratory spaces. Participation was limited to PhD students/candidates to ensure that participants (1) were engaged in benchwork as the primary component of their jobs and (2) were recent college graduates who could make informed reflections about their undergraduate education’s relationship to their current psychomotor laboratory skills. Additionally, all group members were recruited to give consent to enable the recording to continue uninterrupted regardless of who might enter the camera’s range. A laboratory in the recruitment process yielded consent from all group members as well as two participant volunteers. Therefore, that laboratory was selected to proceed with the study. Both participants were second-year organic methodology PhD candidates working on separate projects. Participants received nominal financial compensation for their time. Neither the participants nor their work were previously known to the author.

### Ethics

IRB approval was issued by the author’s institution. Due to the placement of the author and recording equipment within a live workplace, special steps were taken to protect privacy rights and comply with laboratory safety mandates. Workplace actions were not adjusted as a result of the recording, as affirmed by the participants. The video stream from the camera was projected live onto a second screen with remote control capabilities and monitored by the author. If a participant left the designated rooms (e.g., to visit the NMR facility), the camera was temporarily removed, and recording was paused. The participants, participating group, and institution are not named. The PI of the participating group reviewed all images in this article to prevent identifiable visual data from appearing in publication, such as images which might identify the group or the status of their unpublished work. Finally, although grounded theory usually follows a data saturation model to determine when to stop collection, the length of recording was fixed at 2 h/session over 3 sessions/participant with the assumption that 12 h of recording would likely provide sufficient data for a thorough analysis.

### Study Activities and MA Coding

#### 1 Pre-Recording

Participants provided contextual information about their projects, current academic position, and prior laboratory experiences when they volunteered.

#### 2 Recording

**Figure 2:**
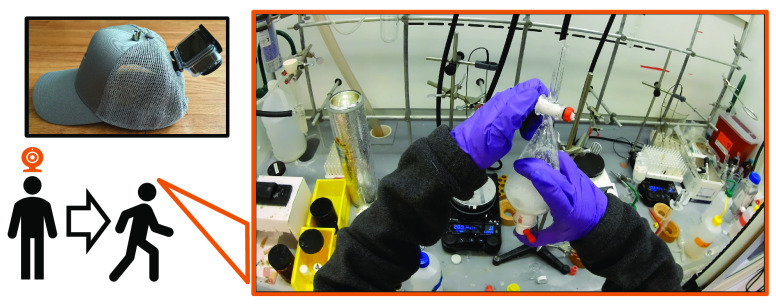
Naturalistic observation was conducted using a first-person action camera worn on a flipped hat.

**Figure 3:**
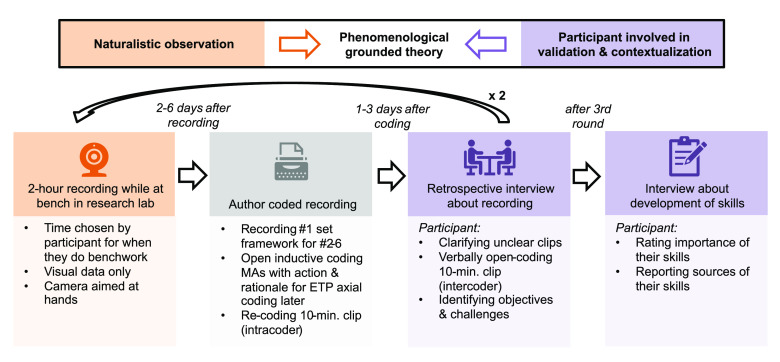
Study activities began with a recording session, followed by coding the recording and conducting a retrospective interview. Naturalistic observation and participant-derived contextualization enabled analysis via phenomenological grounded theory.

Visual data only (no audio) was collected using an action camera attached to a hat on the participant’s head, angled slightly down to capture what their hands were doing (Figure 2). A secondary camera was positioned to film the participant if the action camera was not providing sufficient detail, but this precaution proved superfluous. Each participant underwent three 2-h recording sessions over a few months (Figure 3). Participants proposed time slots for each recording session a few days prior for when they expected to be actively conducting benchwork.

#### 3 Between Recording and Retrospective Interview

Every recording was coded using inductive open basic coding to identify “Meaningful Actions” (MAs, Box 1). The author was surprisingly able to deduce not only the psychomotor skills the participants used but also the underlying rationale for the actions, possibly as a result of personal experience with synthetic research and apparent similarity in synthetic methods between groups and institutions. Therefore, MA codes articulated both the action and the apparent rationale for the participants’ psychomotor actions in sentence-like descriptive text. Video clips had to clear three criteria in order to be eligible for coding (Box 1). Codes could overlap other codes (i.e., the participant could be engaged in more than one MA at a time) and were of varying duration depending on the action. Clips where the action or rationale were ambiguous to the author were marked for later review with the participant. A random 10-min segment of each recording session was coded again to gauge intracoder reliability. Reflective memoing for the first recording of the study was used to develop the MA-ETP framework (Figure 1) for axial analysis based on the emergent themes of “Equipment”, “Technique”, and “Purpose” (ETP) which characterized the ways that participants used their psychomotor skills. Continued reflective memoing and a living codebook were used throughout with new MA codes continuously added to the codebook, and a list of draft ETPs was updated as necessary. The list of ETP codes was finalized and formally assigned to MA codes after completion of all data collection.

Box 1 **Criteria for MA Coding**

1. The MA code must encapsulate a physical action explicitly seen on camera and conducted by the participant themselves.
  - *Example exclusions*: eye movements, mental processes, actions from other people
2. The MA code must encapsulate an apparent underlying rationale for the action.
  - *Example exclusions*: apparently random or idle movements
3. The MA code must appear to contain some form of meaning for the participant’s ability to execute laboratory work, either in terms of the action or the rationale.
  - *Example exclusions*: throwing away paper towels after washing hands, physical manifestations of emotion

Example Meaningful Actions:

- Placing a spatula down carefully so as to avoid contaminating the tip
- Closing the fume hood sash to reduce noise level so labmates do not get annoyed

Example Nonmeaningful Actions:

- Placing a spatula down because there is no reason to hold it anymore
- Fiddling with a knob while waiting for a procedure to finish

#### 4 Post-Recording Interview

A semistructured retrospective interview of up to 1 h was conducted within a week after each recording. Audio was recorded. The semistructured format was selected in order to enable the interviewer to seek clarification on video clips that confused them and to provide a structure in which to elicit the participant’s understanding of the recorded actions. First, the participant was shown a sped-up version of the entire recording via a large projection on the wall to prompt recall. Then, the interviewer highlighted the ambiguous clips, and the participant identified the action and/or explained the underlying rationale. Next, the participant verbally coded a random 10-min sequence to enable analysis of intercoder reliability. The participant was instructed to articulate each MA they felt they noticed, with their attention drawn specifically to focusing on psychomotor skills; the concepts of affective, cognitive, and psychomotor domains were defined to the participant beforehand. They were not provided with a codebook so that their verbal coding could serve as a validation check for the author’s MA coding. The author actively transcribed each MA the participant articulated in their own words and then displayed the list immediately afterward. The participant was asked if they wanted to provide additional context for any MA. Next, the participant was asked contextualizing questions about their overall actions during the recording time slot, such as what the goal of the experiment was. Finally, the participant was asked whether they did anything differently as a result of the recording, both to continually ensure that the study was not intruding on workplace processes and to check for potential Hawthorne effects. Participants affirmed that they did not adjust their actions.

#### 5 Repeat

The coding and retrospective interview process was completed three times for each participant.

#### 6 Between Third and Fourth Interviews

The participant compiled a chronological list of their prior laboratory experiences in preparation for the final interview.

#### 7 Final Interview

The participant was presented with 50 randomly selected MAs coded by the author, and they identified which prior laboratory experience resulted in them learning how to conduct each MA. Then, the participant rated how important they felt it was for incoming organic chemistry graduate students to be able to conduct a selection of 100 random MAs and the ETP items. Finally, they discussed their reasoning for determining the importance of a student knowing those skills.

### Data Processing

Initial background information on the participants and their type of research was collected using either Qualtrics or a paper questionnaire during the recruitment process. After recording, the high-resolution videos were converted to lower resolutions using Xmedia Recode 3.5.4.5. 64 bit (https://www.xmedia-recode.de/en/) to reduce the large file sizes, thereby enabling analysis in MAXQDA. All videos were coded with MA inductive open basic coding in MAXQDA 20.4.1. All interview audio was recorded and then transcribed using intelligent verbatim transcription in MAXQDA. The participants’ verbal coding during the interviews was transcribed verbatim into a spreadsheet and then transported into MAXQDA as MA codes described in their own words. At the final interview, participants filled out paper worksheets (which listed out the sample codes) to rate their perception of the importance of being able to execute their MA and ETP skills and handed in a chronological list of their prior laboratory experiences. After all MA coding was complete in MAXQDA, codes were transferred to Microsoft Excel 365 for ETP axial coding and subsequent analysis. Nominal nonhierarchical two-step cluster analysis with Gower’s dissimilarity measure was executed using SPSS 28.0.1.1.^51,52^ All other data processing was conducted using Excel.

### Data Analysis

Together, the two participants yielded 11.1 h of cumulative recorded time, 6.5 h of which was covered by 2,447 MA codes (Table 1). Third-person naturalistic observation by the author was more effective at identifying MA codes than expected with only 5.5% of coded time requiring review by participants to confirm or provide the action/rationale.

**Table 1:** Descriptive Statistics of Recordings

Recording Sessions
• Six 2-h sessions were recorded over three months (three morning and three afternoon sessions) with two participants (three sessions/participant).
• 11.1 h were recorded and analyzed total.
• 0.5 h were not recorded during sessions due to participants’ temporary departure from the site.

Coded Time
• 6.5 h of recorded time (58.4%) were coded.^a^
• 0.6 h of coded time (9.4%) featured overlapping MA codes.
• 100 clips (0.4 h total = 5.5% of coded time) needed to be reviewed by participants to clarify their action and/or rationale.

Codes
• 2,447 MA codes were assigned (average 408 codes/recording session).
• 303 incidences of MA code overlaps occurred (24.8% of MA codes overlapped with another code).
• MA codes were sorted into one each of 32 Equipment, 30 Technique, and 20 Purpose codes.

a The recorded time without codes often consisted of nonmeaningful actions such as walking around the lab, pausing to think, visually observing changes, or waiting for a procedure to finish.

Unfortunately, calculating inter-rater and intra-rater reliability (or, more properly termed for this type of nominal data, intercoder/intracoder reliability)^53^ is highly challenging for complex naturalistic observational video data featuring overlapping open codes and an evolving, living codebook. Attempts to quantify such comparisons present problematic implications and oversimplification due both to the nature of grounded theory research and the statistical issues arising from a large, complex codebook with multiple rare codes (e.g., prevalence discrepancies).^45,53−57^ However, visual analysis of the coding (see SI) illustrated an apparently high degree of agreement between the participants and author in assigning MA codes, especially after accounting for restrictions that the author operated under in assigning codes which the participants did not (Box 1). The participants sometimes provided more MA codes during their verbal open coding because they could recall cognitive decisions they were making or report on what their eyes were doing behind the camera. They also sometimes mentioned actions that did not appear meaningful (e.g., walking around the lab) seemingly when there was a long gap between MA codes; they appeared to feel a need to fill the silence rather than believing that the actions were truly meaningful. Since the participants were not trained qualitative coders, these discrepancies were not surprising. The author did not correct or interrupt the participants during their coding in order to avoid cuing and to preserve the authenticity of participants’ responses. Accounting for these discrepancies between author and participant coding, the apparent agreement in the visual analysis is encouraging. Since phenomenology is rooted in the acceptance of the participant’s observations as true interpretations of their experiences, the strong agreement also validates the author’s MA coding.

The emerging ETP axial codebook was then finalized with 32 types of equipment, 30 types of technique, and 20 types of purpose that the MA codes were employed in service of (see SI for detailed ETP code descriptions and examples). Each Equipment, Technique, and Purpose code was assigned individually to the MA codes to accurately reflect what the action was, how it was being conducted, and why it was used (Table 2). This careful coding process enabled differentiation for codes that might share one ETP code but not another (e.g., having different purposes despite both being part of a column chromatography technique). Due to the complexity of authentic benchwork, some ETP codes presented the possibility for overlap with other ETP codes. Therefore, some codes were designated as “secondary” to clarify and streamline the coding process whenever a potential conflict was uncovered during ETP coding. An MA code could only be assigned to a secondary ETP code if no other primary code could reasonably be applied (Box 2). Purpose codes were assigned using a “film negative” rationale (drawing upon photography film terminology): the purpose was deduced by considering what the consequences would have been if the action was not undertaken. Six types of Purpose codes presented substantial subcategories referred to here as “optimization”, whereby the participants engaged in actions meant to save them time, effort, or otherwise proactively act to avert a potential issue that would cost them either.

**Table 2:** Examples of ETP Codes

Meaningful Action	Equipment, Technique, Purpose	“Film Negative” Rationale for Purpose
*Example of how Purpose codes can vary with the same Equipment and Technique*		
Labeling column name in automated column’s computer to keep track of conditions	**Equipment**: automated column	Without this step, the record of the column conditions would be lost. Future researchers would be challenged to replicate the same purification procedure.
	**Technique**: column chromatography	
	**Purpose**: enable repetition via records	
Programming pause to fix leaky connection	**Equipment**: automated column	Without this step, product would have been lost through the leak as it came off the column. The obtained yield would have been diminished.
	**Technique**: column chromatography	
	**Purpose**: optimization–maximize product yield	
		
*Examples of the difference between standard Purpose codes and optimization Purpose codes*		
Programming automated column conditions for column	**Purpose**: purify product	Programming the conditions is a necessary step for automatic chromatographic purification. Purification would not have happened without this step.
Adjusting column conditions to finish up faster	**Purpose**: optimization—purify product	Adjusting the conditions saves the researcher time. Purification would still have happened without the step, but it would have taken longer.

Box 2 **Example of Primary vs. Secondary Codes**

- *Primary Equipment Code*: Even though a bump trap is glassware, manipulating a bump trap falls under the primary “rotatory evaporator” Equipment code.
- *Secondary Equipment Code*: Manipulating a separatory funnel did not fall under any primary Equipment codes and so was assigned to “standard glassware”.

## Results

The MA coding documented how the participants were using specific psychomotor skills in the research laboratory environment. The large number of actions and time spent executing them suggest that the participants were effective at using a diverse skillset productively and continuously in the laboratory in pursuit of a variety of goals. Considering that nonmeaningful actions (e.g., walking around, observing changes, consulting with labmates) were not coded, the 6.5 h of coded time out of 11.1 h of recorded time implies that the participants moved quickly from one MA to another with a high level of sustained activity at the bench. Diving deeper into specifics regarding what they were doing and how those actions relate to one another offers interesting insights into how they used their skills to meet the demands of their jobs.

### ETP Code Frequencies

Evaluating the code frequencies illustrates which equipment and techniques were most commonly employed. The most commonly used types of equipment were directly involved in the transfer of chemicals (Table 3). 30.7% of participants’ coded time was spent manipulating solid and liquid transfer tools (e.g., spatulas, weigh paper, syringes, capillary tubes) during 684 distinct incidents. The human body was also frequently employed as a tool itself and often involved in transfer, such as by hitting containers to encourage chemicals to dislodge. Other uses of the body as a tool included warming vials with body heat and applying vigorous force to swirl heterogeneous solutions to encourage dissolution. While the use of records (e.g., personal lab notebook, communal group logs), rotatory evaporators, sundry items (e.g., caps, clips, septa), and standard synthetic glassware (e.g., separatory funnels, Erlenmeyer flasks) featured in another 25.8% of the participants’ coded time, most of the remaining equipment used was interacted with for fleeting time periods. While all the equipment used served vital roles in the participants’ work, this frequency analysis demonstrates an uneven distribution in the materials with which participants most frequently interacted. At the same time, in order to execute their work, participants needed to harness a diverse set of materials for discrete needs, such as the use of a static gun to stabilize a balance’s fluctuating mass measurement or a propane torch to dry glassware for immediate use.

**Table 3:** Frequency of Equipment and Technique Codes

Equipment (*N* = 32)	1°/2°^a^	Count^b^	Duration (%)^c^	Technique (*N* = 30)	1°/2°	Count	Duration (%)
Transfer tools—liquid	1°	470	18.7	Rotatory evaporation	1°	305	10.9
Transfer tools—solid	1°	214	12.0	Cleaning and waste disposal	1°	292	10.4
Human body	2°	301	9.6	Thin-layer chromatography	1°	252	9.8
Records	1°	117	7.6	Notebook management	1°	96	8.1
Rotatory evaporator	1°	198	6.3	Liquid–liquid extraction	1°	263	7.4
Sundry items	2°	127	6.1	Schlenk line operation	1°	103	6.0
Standard glassware	2°	164	5.8	Column chromatography	1°	118	5.5
Computer	1°	118	4.5	Mass transfer—solid	2°	127	5.3
Balance	1°	71	3.5	Laboratory and workspace management	2°	195	4.3
Schlenk line	1°	45	3.3	Mass measurement–solid	1°	92	3.9
Fume hood	1°	162	3.2	NMR analysis	1°	113	3.9
PPE	1°	81	2.0	Inventory management	2°	46	3.6
Calculator	1°	35	2.0	Glovebox operation	1°	48	2.6
Monkey bars and clamps	1°	48	1.9	Mass transfer—liquid	2°	64	2.1
Automated column	1°	35	1.8	PPE management	1°	83	2.1
UV lamp	1°	25	1.6	Air-free mass transfer	1°	39	1.8
Heat gun	1°	12	1.5	Vacuum filtration	1°	23	1.6
Glovebox	1°	27	1.3	Dissolution	2°	49	1.6
Sonicator	1°	12	1.2	NMR sample preparation	1°	28	1.4
Phone	1°	16	1.0	Mass transfer—phase change	2°	8	1.4
Waste collector	1°	41	0.8	Mass measurement—liquid	1°	29	1.4
In-house utilities	1°	33	0.8	Sonication	1°	12	1.2
Stir plate and bar magnet	1°	11	0.8	Solution drying and gravity filtration	1°	11	0.9
Temperature bath	1°	11	0.6	Glassware drying	1°	15	0.7
Column	1°	19	0.5	Temperature bath manipulation	2°	15	0.6
Transfer tools—gas	1°	14	0.5	Literature searching	1°	3	0.4
TLC chamber and stain	1°	18	0.3	Airstream evaporation	1°	7	0.4
Propane torch	1°	3	0.3	Light-sensitive reaction management	1°	3	0.3
Static gun	1°	9	0.2	Solvent system operation	1°	7	0.2
Oven	1°	6	0.2	Chemical drawing software usage	1°	1	0.1
Solvent system	1°	3	0.1				
Fridge	1°	1	0.02				

a Codes are “primary” or “secondary” to account for potential overlap.

b 2,447 Meaningful Actions coded across all recordings.

c Percentage of the cumulative 6.5 h coded, not the 11.1 h recorded.

Concurrently, a frequency analysis of the techniques that this equipment were used to execute illustrates the most common techniques participants employed. Rotatory evaporation, cleaning and waste disposal, and thin-layer chromatography featured prominently, accounting for 31.1% of coded time. Management of personal lab notebooks, liquid–liquid extraction, column chromatography, and transfer of solids represented another 32.3%. The allocation of time to these techniques reflects patterns seen in the equipment frequency analysis, where transfer tools, rotatory evaporators, records, and glassware featured notably. Similarly to the equipment frequency analysis, the technique analysis indicates that participants’ time is not divided evenly among techniques but, rather, is weighted toward specific techniques. At the same time, a larger collection of less frequently utilized techniques continue to fulfill distinct roles. The participants were able to move seamlessly between these equipment and techniques, displaying familiarity with their operation.

These equipment and techniques were together employed in pursuit of a set of ultimate objectives that the participants valued (Table 4 – see SI for detailed definitions and examples). The purposes for much of the participants’ efforts were oriented toward avoiding contamination, purifying desired products out of mixtures, analyzing results, and synthesizing products. However, the participants’ actions also sometimes intersected with the nonexperimental realm (albeit less frequently), such as working to address situations that were causing them personal annoyance without respect to the chemical research itself as well as maintaining good working relationships with their labmates. Participants also spent a quarter of their time engaged in optimization actions, illustrating the degree to which the pursuit of proactive efficiency featured in their efforts. These optimization tactics were especially on display while attempting to avoid contamination, purify a product, and maximize product yield.

**Table 4:** Frequency of Purpose Codes

Purpose (*N* = 20)	1°/2°^a^	Count^b^	Duration (%)^c^	+ *Opt.* Count	+ *Opt*. Duration (%)
Avoid contamination	1°	365	13.7	437	17.3
Purify product	1°	249	11.0	475	20.0
Analyze results	1°	258	10.6	339	12.6
*Optimization - purify product*	*1°*	*226*	*9.0*		
Synthesize product	1°	155	8.1	221	11.6
Enable repetition via records	1°	79	6.3		
*Optimization - maximize product yield*	*1°*	*187*	*5.7*		
Maximize product yield	1°	159	4.9	346	10.6
Enable repetition via measurements	1°	87	4.3	117	5.8
Protect equipment	1°	94	4.0		
*Optimization - avoid contamination*	*1°*	*72*	*3.6*		
Protect people	1°	125	3.5		
*Optimization - synthesize product*	*1°*	*66*	*3.5*		
Enable next step only	2°	38	2.8		
Reduce personal annoyance	1°	98	2.3		
*Optimization - analyze results*	*1°*	*81*	*2.0*		
Maintain work relationships	1°	45	1.8		
*Optimization - enable repetition via measurements*	*1°*	*30*	*1.5*		
Preserve amount of supply	1°	21	1.0		
Preserve product integrity	1°	12	0.3		
All *Optimization* codes together	—	*662*	*25.3*		

a Codes are “primary” or “secondary” to account for potential overlap.

b 2,447 Meaningful Actions coded across all recordings.

c Percentage of the cumulative 6.5 h coded, not the 11.1 h recorded.

### ETP Code Relationships

**Figure 4:**
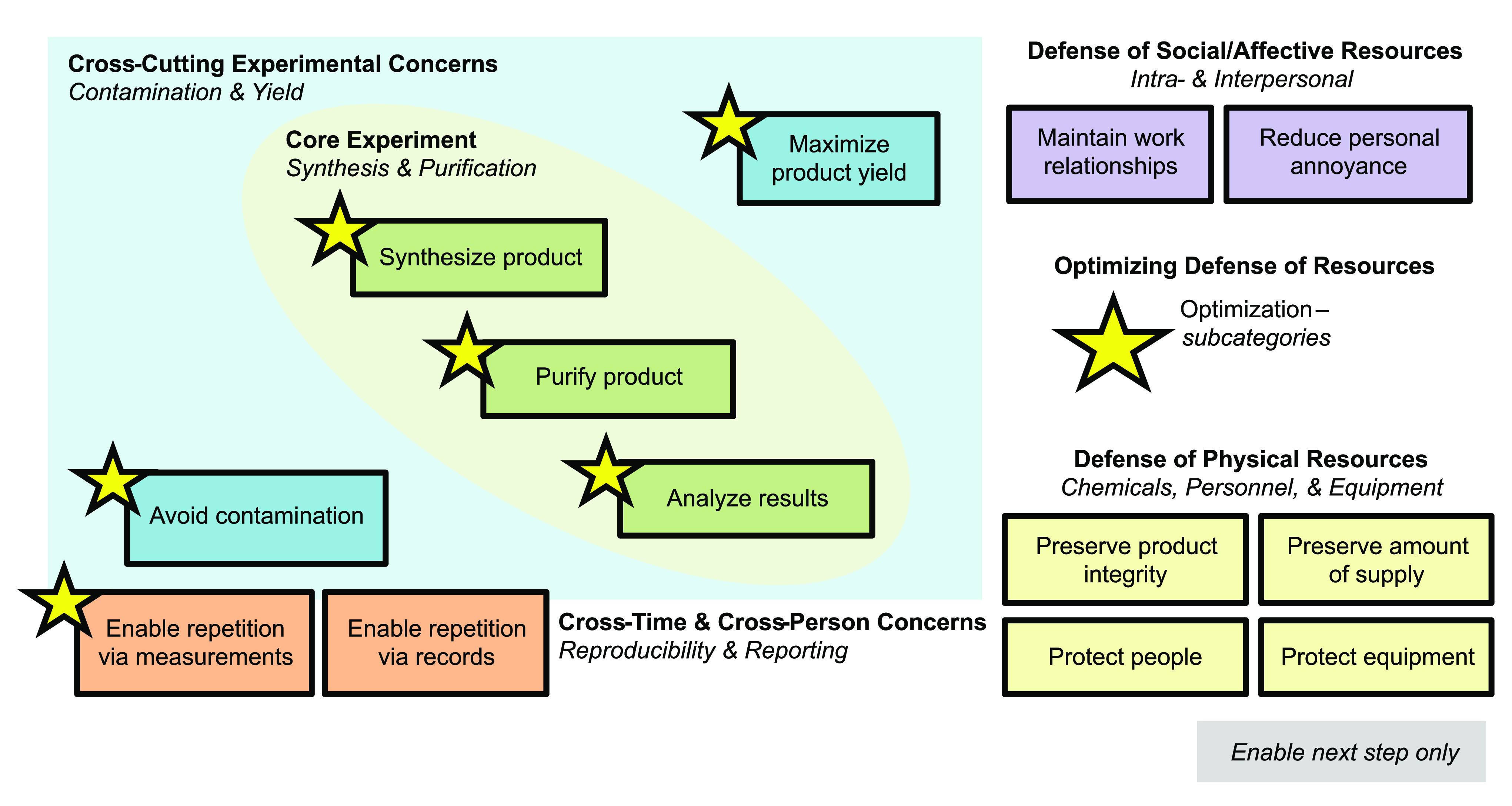
Conceptual map of Purpose codes.

The frequency analyses provide insight into the types of psychomotor skills the participants employed and the materials they interacted with in pursuit of macroscopic purposes. These purposes can be mapped broadly in terms of their relationships to experimental goals or even to social domains (Figure 4). Synthesis, purification, and analysis of experimental results formed the core of participants’ psychomotor actions in the laboratory, but the need to maximize yield and avoid contamination were common cross-cutting concerns among experiments. The participants also operated under the awareness that their work needed to be reproducible by both themselves and other individuals in the future, leading to a prerequisite to both maintain accurate records of their plans and accurately execute those plans as detailed. The centrality of these experimental purposes to the participants’ time in the laboratory helps explain why the participants focused on optimizing their efforts in these areas. Optimization tactics can be viewed as a form of defensive behavior to guard against wasting precious physical resources (e.g., loss of material), time, and affective resources (e.g., patience to wait for a process to finish). The other purposes which were not tied as directly to the execution of the experiment themselves can also be viewed as defensive in nature: the defense of social (e.g., relationships with labmates) or other affective resources (e.g., emotional state) and the defense of physical resources (e.g., a chemical prone to degradation, personnel who could be injured, specialized equipment that would be expensive to replace).

**Figure 5:**
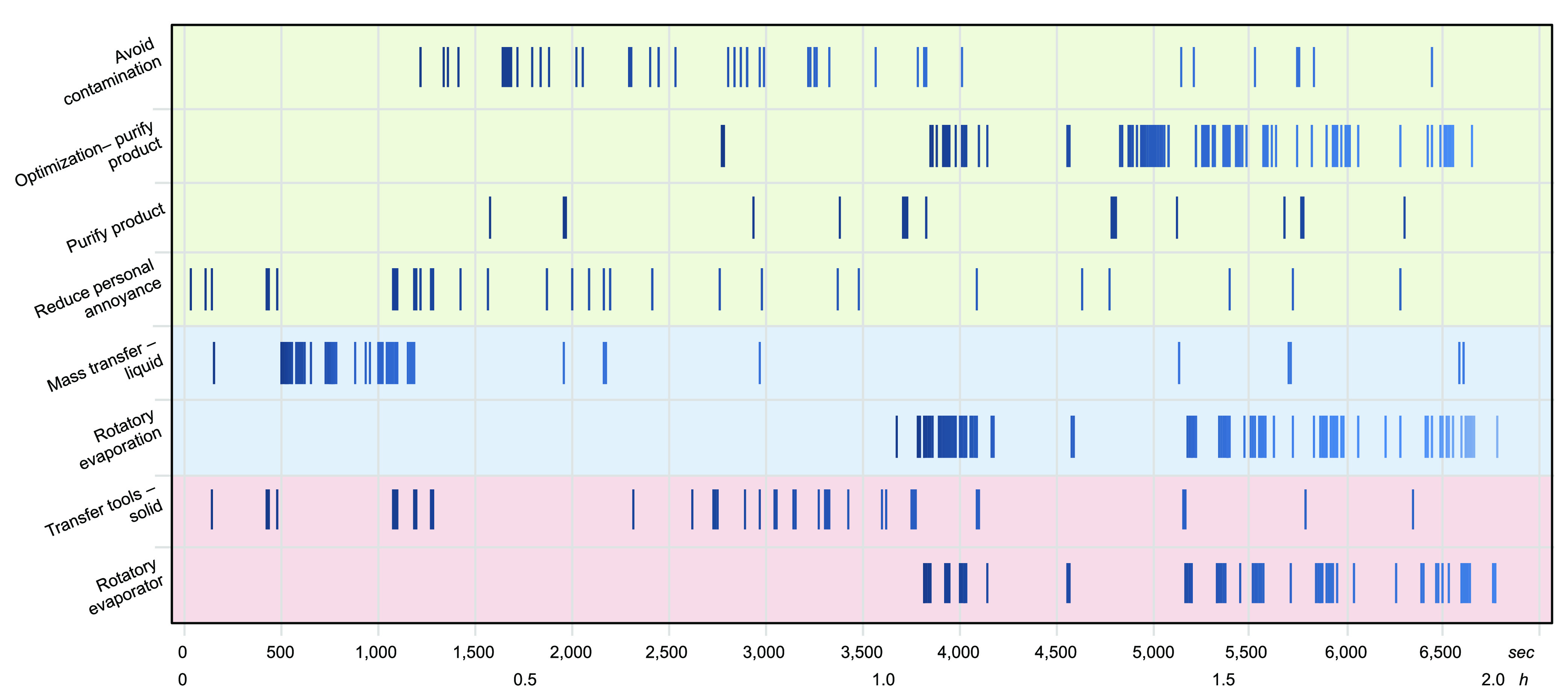
Example temporal analyses of selected Equipment (red, bottom), Technique (blue, middle), and Purpose (green, top) codes in one full recording session.

This concept mapping helps to illustrate why participants did what they did but does not, by itself, explain *how* the techniques and equipment highlighted earlier were used to this effect. As seen in temporal analyses of selected ETP codes (Figure 5), the participants deployed their skills in complex ways. In the sample temporal analyses displayed, some ETP codes are concentrated in time (e.g., liquid mass transfer), whereas others (e.g., the use of solid transfer tools) are distributed widely. The use of flagship equipment such as the rotatory evaporator does not fully account for all the equipment used during its namesake technique, rotatory evaporation. Steps taken to optimize purification often immediately followed the pursuit of purification but not always. These temporal analyses demonstrate the complexity of the laboratory work and lack of surface-level discernible patterns in the interplay between equipment, techniques, and purposes.

**Figure 6:**
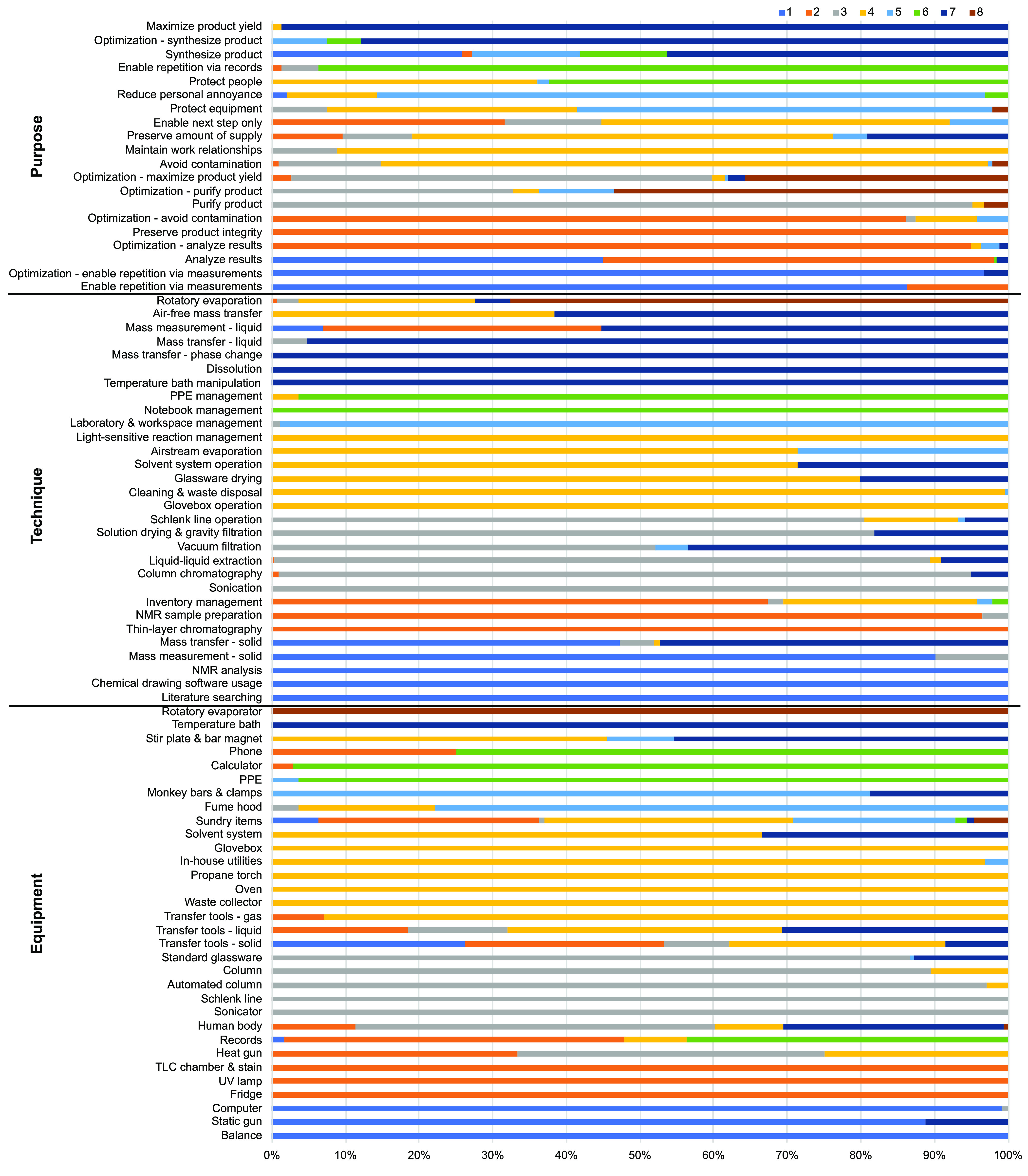
Distribution of Equipment, Technique, and Purpose codes by cluster (cluster legend top right corner).

Cluster analysis, however, helps illuminate the interactions on a deeper level (Figure 6 – see also supplemental figures in SI). Cluster analyses were modeled between ETP codes until a maximum average silhouette value was reached at *k* = 0.2670 with eight clusters (see SI). Due to the descriptive nature of cluster analyses, there is no formal cutoff for determining the “significance” of an exploratory, descriptive cluster analysis.^51,52^ Instead, uniformity in items’ silhouette values, a high average silhouette value, and perceived reasonableness of the resulting clusters comprise a set of approaches often considered in evaluating a cluster analysis. Since the presence of overlapping codes between clusters was expected based on the complex nature of laboratory work, some overlap in the clusters is not necessarily seen as an indicator of poor clustering for this data.

#### Cluster Descriptions

Refer to Figure 7:

- **Analyzing NMRs and Preparing Chemicals:** Cluster 1 demonstrates that using a balance, computer, static gun, and solid transfer tools are often associated with chemical drawing software usage, literature searching, solid mass measurement, solid mass transfer, and NMR analysis techniques. These equipment and techniques are employed when working to analyze results, measure accurately while employing optimization tactics, and synthesize a product. *In sum, cluster 1 describes both how participants carefully prepared the chemicals for their upcoming reactions and later analyzed the final spectroscopic results*.
- **Making NMR Samples and Running TLCs:** Cluster 2 demonstrates that using a fridge, heat gun, the human body, phone, records, sundry items, a TLC chamber and stain solution, liquid and solid transfer tools, and a UV lamp are often associated with inventory management, liquid mass measurement, NMR sample preparation, and thin-layer chromatography techniques. These equipment and techniques are employed when working to analyze results while employing optimization tactics, enable necessary next steps in a procedure, employ optimization tactics while avoiding contamination, and preserve a desired product’s integrity. *Cluster 2, therefore, describes how participants efficiently analyzed results using TLC and prepared NMR samples, all while keeping accurate records of this work and handling related chemical storage*.
- **Purifying Crude Mixtures:** Cluster 3 demonstrates that using an automated column machine and its associated column, a heat gun, the human body, a Schlenk line, sonicator, and standard glassware are often associated with air-free Schlenk line operation, column chromatography, liquid–liquid extraction, solution drying and gravity filtration, sonication, and vacuum filtration techniques. These equipment and techniques are employed when working to avoid contamination, employ optimization tactics while maximizing yield, and purify a product while employing optimization tactics. *Cluster 3 describes how participants purified desired products out of crude mixtures while trying hard to be efficient throughout*.
- **Using Scientific Equipment and Room Infrastructure:** Cluster 4 demonstrates that using a heat gun, fume hood, glovebox, the human body, in-house utilities (e.g., sink water line, ventilated cabinets), oven, propane torch, solvent system, sundry items, stir plate and bar magnet, gas/liquid/solid transfer tools, and waste collector are often associated with air-free mass transfer, airstream evaporation, cleaning and waste disposal, solvent system operation, glassware drying, glovebox operation, inventory management, light-sensitive reaction management, and rotatory evaporation techniques. These equipment and techniques are employed when working to avoid contamination, enable the next procedural step, maintain work relationships, preserve materials, and protect people and equipment. *Cluster 4 describes how participants used a variety of scientific equipment and specialized infrastructure to maintain social and physical resources (e.g., chemical stock supplies, equipment, personnel safety) in prime operating condition and ready for experimental use*.
- **Working in a Fume Hood:** Cluster 5 demonstrates that using a fume hood, monkey bars and clamps, and sundry items are often associated with airstream evaporation and laboratory and workspace management techniques. These equipment and techniques are employed when working to protect equipment, reduce personal annoyances, and synthesize a product. *Cluster 5 describes how participants worked within the confines of a fume hood to optimize the setup of their workspace and to deal with recurring annoyances associated with fume hoods (e.g., sash alarms)*.
- **Staying Safe and Using a Notebook:** Cluster 6 demonstrates that using a calculator, phone, personal protective equipment (PPE), and records are often associated with notebook and PPE management techniques. These equipment and techniques are employed when working to enable procedure repetition and protect people. *Cluster 6 describes how participants utilized PPE for their own personal safety and kept appropriate records of their work to enable future reproducibility*.
- **Setting Up and Monitoring Reactions:** Cluster 7 demonstrates that using the human body, monkey bars and clamps, solvent system, stir plate and bar magnet, temperature bath, and liquid transfer tools are often associated with air-free mass transfer, dissolution, solvent system operation, glassware drying, liquid mass measurement and mass transfer, phase change transfer manipulations (e.g., manipulating the phase of a chemical to move it as a liquid instead of as a solid or vice versa), solid mass transfer, temperature bath manipulation for reactions, and vacuum filtration techniques. These equipment and techniques are employed when working to maximize yield and synthesize a product while employing optimization tactics. *Cluster 7 describes how participants efficiently assembled the materials for reaction set-ups, started reactions, and made necessary adjustments to maintain desired conditions throughout the reaction*.
- **Rotatory Evaporating:** Cluster 8 demonstrates that using a rotatory evaporator is often associated with rotatory evaporation techniques. These equipment and techniques are employed when working to employ optimization tactics while maximizing yield and purifying a product. *Cluster 8 describes how participants’ interactions with a rotatory evaporator were primarily intended to optimize the expenditure of their time and effort during the overall purification process*.

**Figure 7:**
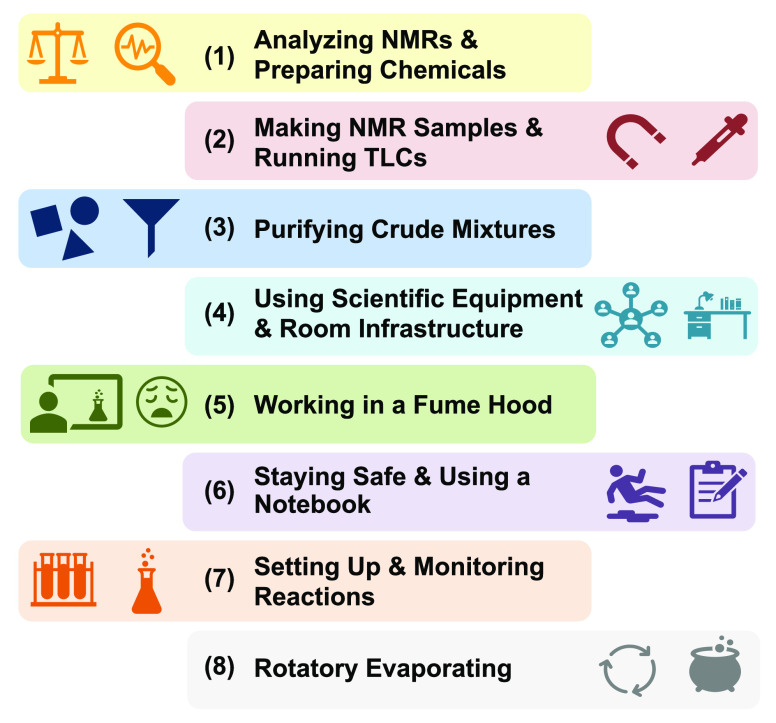
Characteristics of clusters.

### Perceptions of Value of Skills

Together, the eight clusters describe the complex ways in which the participants employed their equipment and techniques in pursuit of their overall purposes. To probe how the participants themselves perceived these skills and the role that the skills played in their ability to conduct their doctoral research, the participants were asked to rate how important it would be for an incoming organic chemistry graduate student to be able to use each equipment item, execute each technique, and accomplish each purpose. Participants were shown the overall list of ETP codes and a list of 100 randomly selected MA codes (without the corresponding ETP codes shown). They marked items as being either essential (score = 1), very helpful but not essential (2), good to know (3), or not important (4) for an incoming organic chemistry student to be able to do to be successful in graduate research.

Participants’ averaged responses indicate that the participants overwhelmingly held these skills in high regard (Table 5). Approximately half of the items in each ETP category received unanimous “essential” scores. One participant tended to rate the remaining codes lower than the other participant as a reflection of their belief that “these are things that really help you if you can come in and be independent of them and understand what’s happening, but they are pretty easy to pick up along the way” (Participant B). Only two ETP codes received an average face value score of 3 or lower: “phone” (Equipment) and “airstream evaporation” (Technique). The phone was mostly used to take pictures of results or the notebook for reference later, and airstream evaporation was utilized on a few occasions to reduce the solvent volume in a vial by applying nitrogen gas flow. Otherwise, participants agreed on the important role that most of their demonstrated skills play in achieving research success.

**Table 5:** Perceived Value of Lab Skills^a^

Equipment	Face Value Score^b^	Avg. Item Score	Technique	Face Value Score	Avg. Item Score	Purpose	Face Value Score	Avg. Item Score
Calculator	1.0	1.0	Mass measurement - liquid	1.0	1.0	Analyze results	1.0	1.0
Computer	1.0	1.0	NMR analysis	1.0	1.0	Enable repetition via records	1.0	1.0
Temperature bath	1.0	1.0	NMR sample preparation	1.0	1.0	Protect equipment	1.0	1.0
Transfer tools - liquid	1.0	1.3	Notebook management	1.0	1.0	Enable repetition via measurements	1.0	1.3
Balance	1.0	1.5	Solution drying and gravity filtration	1.0	1.0	Avoid contamination	1.0	1.5
Records	1.0	1.5	Mass transfer - solid	1.0	1.4	Protect people	1.0	2.4
Fume hood	1.0	1.7	Mass transfer - liquid	1.0	1.4	Purify product	**1.5**	**1.3**
Transfer tools - solid	1.0	1.7	Thin-layer chromatography	1.0	1.4	Synthesize product	**1.5**	**1.4**
Standard glassware	1.0	2.0	Cleaning and waste disposal	1.0	1.5	Maximize product yield	1.5	1.7
Stir plate and bar magnet	1.0	2.0	Mass measurement - solid	1.0	1.7	Maintain work relationships	1.5	2.0
Column	1.0	2.5	Column chromatography	1.0	1.9	Preserve amount of supply	1.5	2.5
PPE	1.0	2.5	PPE management	1.0	2.3	Preserve product integrity	1.5	—
Fridge	1.0	—c	Glassware drying	1.0	3.0	Reduce personal annoyance	2.5	2.8
Monkey bars and clamps	1.0	—	Liquid–liquid extraction	1.0	—	Optimization - avoid contamination	—	1.0
TLC chamber and stain	1.0	—	Literature searching	1.0	—	Optimization - analyze results	—	1.5
Waste collector	1.0	—	Rotatory evaporation	**1.5**	**1.1**	Optimization - maximize product yield	—	1.5
Rotatory evaporator	**1.5**	**1.0**	Vacuum filtration	1.5	1.7	Enable next step only	—	1.7
Human body	1.5	2.0	Dissolution	1.5	1.8	Optimization - purify product	—	1.9
Schlenk line	1.5	2.0	Mass transfer - phase change	1.5	2.0	Optimization - enable repetition via measurements	—	2.0
UV lamp	1.5	2.0	Laboratory and workspace management	1.5	3.0	Optimization - synthesize product	—	2.0
Sundry items	1.5	2.2	Air-free mass transfer	1.5	—			
Heat gun	1.5	—	Chemical drawing software usage	1.5	—	*“Optimization” presented to participants as one category*^d^	1.5	1.8
Oven	1.5	—	Solvent system operation	1.5	—			
Sonicator	1.5	—	Light-sensitive reaction management	**2.0**	**1.0**			
Glovebox	**2.0**	**1.0**	Temperature bath manipulation	**2.0**	**1.0**			
Transfer tools - gas	**2.0**	**1.0**	Glovebox operation	**2.0**	**1.6**			
In-house utilities	**2.0**	**1.5**	Schlenk line operation	2.0	2.1			
Solvent system	2.0	—	Sonication	2.0	—			
Static gun	2.0	—	Inventory management	**2.5**	**2.2**			
Automated column	**2.5**	**1.0**	Airstream evaporation	**3.0**	**2.0**			
Propane torch	2.5	—						
Phone	4.0	—						

a The participants’ scores for each item were averaged. Participants rated items by importance for an incoming synthetic graduate student to be able to do (1 = essential; 2 = very helpful but not essential; 3 = good to know; 4 = not at all important).

b Participants were asked about each item’s importance explicitly (“face value score”) and about random MA codes representing a sample variety of categories, later averaged together per category (“avg. item score”).

c Due to the large number of codes and realistic constraints on participants’ time, participants were not able to rate the importance of MA codes for every ETP code. Participants were given 100 randomly selected MA codes to rate, and those sample items enabled composite scores for the ETP codes shown here.

d It was thought that participants might struggle to meaningfully distinguish between optimization categories without in-depth training with the codebook, and so they were presented with “optimization” as one single category.

Of note, however, is the trend where some individual MA codes received lower composite scores than their ETP codes did at face value (bolded). Both participants explained that, while they may highly value the skill represented by an overall ETP code, there can be different ways of executing an ETP skill:

*Participant B: There, it was more things that everyone has their own way of doing this. You’ll figure out a way to do this. The destination is more important than the journey, so it doesn’t matter if you’re doing it the same way that I’m doing it*.

Therefore, participants tended to give lower scores for the essentiality of certain specific MA codes because they perceived potential flexibility in how another person might execute an equivalent skill. However, there were multiple instances where the MA codes were rated higher than the ETP codes they represented, possibly reflecting the expressed belief that sometimes there is only one right way to execute a skill and that proper execution is essential in such cases:

*Participant A: Like, maybe using a glovebox is very helpful for students to do if they enter, but not every reaction needs a glovebox. But for the specific [meaningful action item], if they don’t purge the glovebox, that could harm the glovebox irreparably*.

### Perceptions of Origination of Skills

Participants’ beliefs that most of their skills are essential or very helpful for incoming graduate students also reflects on their own educational histories and preparations for PhD-level research. Both participants participated in chemical research during college, took a set of introductory and upper-level laboratory courses, and participated in research experiences outside of academic laboratories (Table 6). Both specialized in organic synthesis as undergraduates and in organic methodology as PhD students, but they also pursued additional experiences in other subfields of chemistry before entering their PhD program. When participants were asked to identify when they learned how to do a random selection of 50 MA codes (Table 7), they overwhelmingly reported learning the skills before entering the PhD program. Their PhD experiences sometimes served to refine certain skills, but the participants reflected that they were using equipment and conducting techniques without substantial differences from their undergraduate training via formal laboratory coursework and undergraduate research experiences. Both participants, however, commented that they have encountered situations where they themselves created a solution to a psychomotor challenge they were encountering, rather than having learned the solution somewhere else:

**Table 6:** Participants’ Laboratory Experiences

Participant’s Context	Participant A	Participant B
*Year in PhD program*	2nd	2nd
*Pathway from undergrad*	NIH postbaccalaureate for 2 years	Immediate transition to PhD program
*Degree before PhD program*	Master’s	Bachelor’s
*Type of undergrad institution*	R1, USA	R1, USA
*Types of lab experiences before PhD program*	• Cookbook lab	• Cookbook lab
	• Inquiry lab	• Inquiry lab
	• Research lab	• Course-based research
		• Research lab
*Overview of STEM laboratory experiences before PhD program*	• Intro undergrad biology, honors physics, and majors organic chemistry lab courses	• Intro undergrad physics, biology, and honors general chemistry lab courses
	• Upper-level lab courses in organic, inorganic, analytical, and physical chemistry	• Upper-level lab courses in analytical and physical chemistry
	• Research in organic synthesis, inorganic synthesis, and radiochemistry in academia and NIH	• Research in organic synthesis and medicinal chemistry in academia and industry
*Focus of PhD research*	Organic methodology (mostly solo projects)	Organic methodology (mostly solo projects)

**Table 7:** Perceived Sources of Lab Skills

Experience Resulting in Lab Skill^a^	Initially Learned^b^	Improved/Adjusted	Improved/Adjusted (again)
**Participant A**			
High school	1		
Organic chemistry I laboratory course	8		
Synthetic organic/inorganic chemistry research laboratory (undergraduate)	10	5	
Advanced organic chemistry laboratory course	3		
Synthesis and radiochemistry research laboratory (NIH postbaccalaureate)	10	5	
Synthetic organic chemistry research laboratory (doctoral)	7	7	1
**Participant B**			
“Common sense”		1	
General chemistry I laboratory course	6		
Biology I laboratory course		1	
Synthetic organic chemistry research laboratory (undergraduate)	31	2	
Medicinal chemistry research laboratory (industry internship)	4		
Synthetic organic chemistry research laboratory (doctoral)	8	1	

a Organized in descending chronological order for when the participant started the experience.

b For 50 random Meaningful Actions each from a list of actions that each individual participant demonstrated.

*Participant B: I think there is a lot of MacGyvering that goes into labwork or arts and crafts. Making different contraptions to accomplish what you need to get done. And that’s been really helpful for me and I guess the fun part of research. Oh, I need to evaporate solvent from these two small vials, but I don’t have an adaptor directly for my line. And then figuring out what kind of things you can put together to make the contraption you need*.

### Role of Automaticity

Finally, it is worthwhile to note a theme about automaticity which emerged over the course of the eight interviews. When participants were asked to discuss the rationale for their specific actions or their overall experimental goals, they often mentioned either challenges they encountered or their observation that the session “went smoothly”. On several occasions, participants noted that, after encountering a challenge to their psychomotor actions in the laboratory, they made a decision to either redo the sequence of previous actions (e.g., recollect bumped material and attempt rotatory evaporation again), adjust their standard operating protocol moving forward (e.g., add in a different solvent to the liquid–liquid extraction to try to improve solubility), or add in a supplemental technique to address the challenge (e.g., try to sonicate a silica gel mixture to achieve dissolution before attempting rotatory evaporation in preparation for dry loading a column). In the absence of an unexpected challenge, participants appeared to view most of their psychomotor actions as routine which, coupled with the high level of physical productivity displayed over the 11.1 h of recorded time, suggests that the participants had achieved psychomotor automaticity regarding the use of their equipment, execution of techniques, and pursuit of purposes. Skills which were perceived as requiring a certain degree of familiarity and automaticity also tended to be rated as essential skills:

*Participant B: [The essential skills are] mostly the skills that we use everyday that typically we maybe don’t think exactly about all of the reasoning behind*.

When they encountered a disruption to their automatic processes, however, they were forced to reevaluate and engage deliberately in determining the best next steps:

*Participant A: There is a lot of muscle memory with certain lab techniques, so good researchers will be able to complete these techniques quickly without hesitation. However, they must also be able to change course and re-assess if something goes wrong, such as an unexpected heat, color change, precipitation*.

Automaticity with cognitive skills such as arithmetic have previously been shown to be helpful with general chemistry performance.^58^ The interplay of psychomotor automaticity with chemical research competencies likely deserves additional attention in future studies.

## Discussion

This paper sought to illuminate how chemists use their psychomotor skills when conducting bench research and how they learned those skills. Through phenomenological grounded theory case studies with naturalistic first-person recordings and retrospective interviews, a MA–ETP framework was established through which to characterize psychomotor actions in laboratories and enable future development of NGLSs.

### Implications

From a methods standpoint, third-person naturalistic observation was more effective than expected in a complex workplace. The discovery of the MA-ETP framework lent effective and much-needed structure to the coding process. Future laboratory-based research could employ this framework when dealing with similarly large volumes of complex data about the physical realm. Additionally, the high degree of agreement in coding could indicate that synthetic chemical methods are similar between groups and institutions. If so, such commonalities could perhaps empower undergraduate laboratory experiences to expose students to many of the equipment and techniques in use in graduate-level synthetic laboratories.

Interviews with faculty have already shown that learning practical techniques is a key focus of some laboratory courses, especially in organic chemistry.^21,59^ The chemist participants had learned a range of relevant psychomotor skills before entering their PhD program, mostly as a result of prior research experiences as well as laboratory courses. Descriptive statistics and temporal analyses of the recordings indicate that participants engaged frequently with their psychomotor skills, maintaining a high level of sustained activity. The participants’ comments about automaticity and essentiality scores highlight the value they placed on entering their PhD program with a suite of familiar skills as well as being able to adjust their actions when necessary. They displayed their own abilities to deploy skills seamlessly, as evidenced by their high degree of psychomotor activity and ability to weave diverse skills together throughout time and clusters. Their activity level and educational chronologies indicate that their prior laboratory experiences provided relevant training opportunities and that psychomotor automaticity played an important role in their current work. They reported positively on the need for students to attain similar skills before entering graduate school with the caveats that specific skills may differ in whether there is flexibility in the exact methods used. While some of their equipment and techniques were employed often (e.g., transfer tools, rotatory evaporation) in pursuit of their ultimate purposes (e.g., avoiding contamination), many of the ETP codes appeared infrequently (e.g., static gun, reduce personal annoyances). However, the objects of these infrequent ETP codes still played crucial roles.

These observations imply that undergraduate synthetic chemistry students would be well-served by being exposed to a variety of equipment and techniques and being given opportunities to practice and refine their physical execution. Students could also benefit from being coached on how to engage in practical problem-solving (or “MacGyvering”) to generate solutions to physical challenges. Moreover, the role of proactive efficiency-seeking featured positively and prominently. This focus on efficiency provides a contrast to common anecdotal complaints among undergraduate educators that a major focus of students in laboratory courses is how quickly they can manage to finish an experiment (and undergraduates’ stress about finishing in time^60^). The majority of the participants’ recorded time was occupied by coded psychomotor activity of which most was in service of the experimental domain but also featured defensive concerns about social, affective, and physical resources. The quests for purity (both in terms of achieving purity and maintaining it) and efficiency constituted the largest demands on their time. Time is, after all, a limited, nonrenewable resource, and the participants displayed an awareness of many ways they could work to safeguard their time. The parallels between the participants’ and undergraduate chemistry students’ attention to time may indicate that efficiency is ultimately a valuable learning objective itself (albeit not the only learning objective). This observation dovetails with the theme of automaticity: automatic skills can be deployed efficiently. Undergraduate educators may find themselves working in the future to balance how to train students such that they achieve automaticity with psychomotor skills but also possess practical problem-solving skills and understand the conceptual underpinnings well enough to adjust to unexpected circumstances.

### Limitations

A study involving two participants as case studies provides strength in depth but not in breadth. Especially since the participants were in the same research group, the equipment, techniques, and purposes shown here may not reflect the average experience in other synthetic chemistry research laboratories. Since the participants were in their second year of their PhD program, the participants’ psychomotor competencies may have substantially improved since they entered the program, despite their recollections of learning a greater proportion of their skills beforehand. Additionally, academic graduate research laboratories represent only one type of laboratory workplace. In general, more research needs to be done with industrial, governmental, and other types of chemists to elucidate their experiences and how those experiences relate to their earlier formal educational training.

The participants reported (when explicitly asked at the end of each interview) that they did not change their actions in the laboratory as a result of the recordings. The participants were aware from the explicit study recruitment information that the study was not evaluating them or their experimental results but instead was an exploration of how bench research happens inside synthetic chemistry laboratories. Presumably, if the participants felt pressure to behave in a specific way (e.g., due to a Hawthorne effect), they would have wanted to increase their productivity and/or avoid mistakes while being recorded. However, they were told that it was completely ok to make mistakes (e.g., spills, breakages), take down time, or struggle with their experiments because seeing them go through the process of stopping to think or address challenges was important, authentic data for the observer. They were told that the purpose of filming them was to help improve undergraduate laboratory experiences and to understand how advanced researchers like themselves actually do their work. The participants’ repeated affirmations that they did not change their actions as a result of the recording and their readiness to self-identify (during the interviews) challenges they experienced subsequently lends support to the authentic representativeness of the data for the benchwork-intensive parts of their workday. The interviewer adopted a casual, conversational attitude during these discussions to normalize challenges and mistakes. However, it is also possible that the presence of recording equipment adjusted how other people in the laboratory behaved: the prevalence of social interactions may have been lower as a result of the visible camera on the participants’ heads. While investigating social interactions was not a purpose of this study, it is relevant to keep in mind.

Of course, there are other activities that participants engaged in to conduct their doctoral research; this study did not purport to focus on the cognitive or affective domains of their jobs. The recording time slots were ultimately chosen by the participant in anticipation of when they would be working at the bench, so the high degree of physical action illustrated in the data should not be construed as comprising their overall workday. Some of the ETP codes may also appear, on the surface, to be underrepresented due to the need to employ a primary-secondary hierarchy for the sake of consistent coding. For example, the participants handled glassware more often than for 5.8% of the coded time. The bump trap of a rotatory evaporator, the Schlenk line, and other glassware that constituted integral components of primary Equipment codes were not represented in the “standard glassware” secondary code.

Additionally, the nature of the research itself presents limitations in the data analysis process. Besides the participants who engaged in validative open coding, the author was the sole trained coder. Literature generally does not support the use of inter-rater/intercoder reliability for complex naturalistic observational video data featuring overlapping open codes and a living codebook, and literature is also hesitant about applying reliability analyses in qualitative grounded theory research due to the importance of the reflective memoing and iterative, cyclical nature of analysis.^53,55−57^ Additionally, calculations of reliability coefficients in situations with large data sets involving many codes with wildly different prevalences can be problematic because they might present artificially low values as a result of mathematical demands. While the SI provides detailed notes and applied examples of the codebook for precisely these reasons, being able to conduct a reliability analysis would lend greater support to the credibility of the data analysis and subsequent conclusions.

Finally, this data provides an idea for what the product of undergraduate education could look like but does not offer a pathway for achieving it. The participants themselves identified that there can be variability in how skills are executed, and the author noticed during the recordings that they did not always conduct the same technique in the same way as the other participant, despite being in the same group. The questions of how to best teach students psychomotor skills, automaticity, and practical problem-solving remain open. Finally, most students in undergraduate chemistry courses do not proceed to doctoral chemistry programs; their learning objectives in laboratory courses may not necessarily align with that of molding students into future graduate student researchers.

## Conclusion

Psychomotor skills are crucial in the laboratory since it is a physical, hands-on workplace. As the DBER community and educators across the world work to improve STEM education, the psychomotor domain deserves explicit attention in investigating how to promote effective, authentic learning. These case studies offer unique insight into the laboratory as a dynamic, challenging environment that undergraduate education seeks to prepare students to enter. Viewing the psychomotor domain in terms of the *what-how-why* MA-ETP framework discussed herein may guide future research and educational innovations. Sharing a consensus across chemistry education on how to view and evaluate the types and purposes of psychomotor actions in laboratory settings could set the stage for innovations deliberately cultivating students’ capabilities in these areas. By elucidating the role that psychomotor skills play in the competencies of laboratory-based chemists, DBER can lead to the development of relevant NGLSs for future generations of chemistry students.

## Supporting Information Available

Codebook with Illustrative Examples (PDF)
Video Clip with Example Coding (MP4)
Cluster Analysis (PDF)
