# Supplementary material for: A Day in the Life: Characterization of Doctoral Bench Research in Synthetic Chemistry Using Phenomenological Case Studies
Source: J Chem Educ. 2022 Dec 28;100(2):442–58. doi: 10.1021/acs.jchemed.2c00809 (PMC9933917; doi:10.1021/acs.jchemed.2c00809)

Supporting Information: Codebook with Illustrative Examples

A Day in the Life: Characterization of Doctoral Bench Research in Synthetic Chemistry using Phenomenological Case Studies

Elizabeth W. Kelley*
Chemistry Department, University of Chicago, 5735 S Ellis Ave, Chicago, IL, 60637, USA
*ewkelley@uchicago.edu

CONTENTS

- Codebooks for Equipment, Technique, and Purpose codes p. 2-20
- Textual analysis of example video clip p. 21-22
- Temporal analysis of qualitative inter- and intra-coder reliability for coding Meaningful Actions p. 23

After data collection, Meaningful Actions (MA) were classified with one code each from the Equipment, Technique, and Purpose list.

Some codes were deemed "secondary", meaning that they were only assigned if no other "primary" code applied. This clarified the coding process for situations where potential overlap might cause inconsistencies during coding.

Primary vs. Secondary examples:

- Manipulating a bump trap did not fall under the secondary "standard glassware" Equipment code but rather the primary "rotatory evaporator" Equipment code.
- Manipulating a separatory funnel did not fall under any primary Equipment codes and so was assigned to "standard glassware".
- Most chemical additions could technically be classified as a "dissolution" Technique, so an action was only classified as "dissolution" if the action could not be classified under other primary Technique codes (for example, "sonication").

<u>Meaningful Action</u> = a specific, basic psychomotor action related to accomplishing a task while working in a laboratory

- Coded using sentence-like descriptive text articulating the action and the apparent rationale

<u>Equipment</u> = a tangible object, its integral components, and/or a set of highly related objects used in conjunction with the meaningful action to accomplish a technique or purpose

<u>Technique</u> = a cohesive sequence of meaningful actions to complete a specific procedure in a discrete span of time or a series of repeated meaningful actions spread out across time to meet a common objective

<u>Purpose</u> = an objective that possess value in the macrocosm of the "real-world", regardless of a specific technique or equipment

Example photos are cropped, grainy, and zoomed in both to focus on the applicable MA and to exclude potentially identifying information about the participants. An example video is also provided in the SI to demonstrate the coding process.

Table S1. Codebook for Equipment.

#	Equipment Code	Definition	Primary / Secondary	Applicable MA Example	Image of Applicable MA Example	NOT an Applicable MA Example
E1	Automated column	operating the automated column machine – does not include the column itself	Primary	Adjusting column conditions to finish up faster	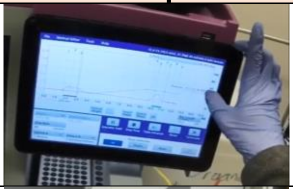	Packing frit to be flush<br>- Column
E2	Balance	operating the balance machine – does not include the associated transfer tools (e.g., spatulas, syringes)	Primary	Fixing crooked balance tray to measure mass	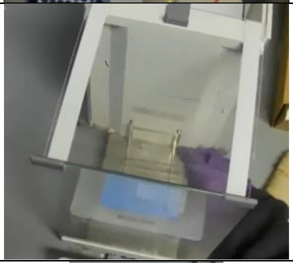	Using static gun to stabilize balance measurement<br>- Static gun
E3	Calculator	operating a calculator	Primary	Calculating amounts to determine starting materials	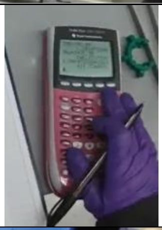	Drawing in chemical drawing software to get molar mass for calculations<br>- Computer
E4	Column	manipulating the components of a column (e.g., frits and attachments) – does not include the automated column machine	Primary	Packing frit to be flush	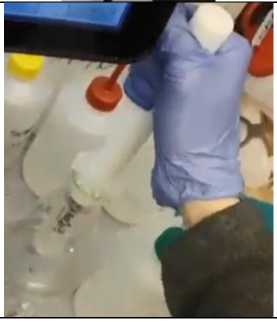	Adjusting column conditions to finish up faster<br>- Automated column

E5	Computer	operating a computer and scientific software	Primary	Calibrating NMR integrals' proton count	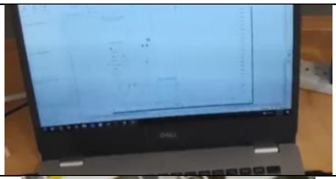	Removing safety glasses while working at computer<br>- PPE
E6	Fridge	using a fridge	Primary	Storing compound in fridge to prevent decomposition	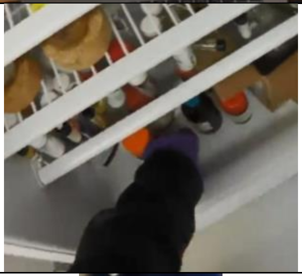	Labeling container to keep track of compound identity<br>- Records
E7	Fume hood	operating a fume hood and its built-in utilities (e.g., vacuum line, air line)	Primary	Decreasing air flow to reduce loud annoying sound	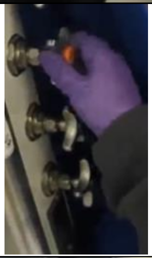	Switching line needle to enable a greater flow of inert gas<br>- Schlenk line
E8	Glovebox	operating a glovebox and its built-in components (e.g., antechamber, glovebox gloves)	Primary	Evacuating glovebox chamber to ensure a tight door seal	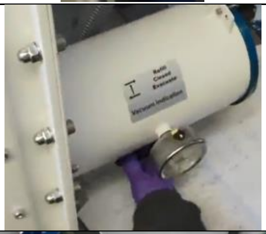	Taking notes to enable distant work at glovebox<br>- Records
E9	Heat gun	operating a heat gun	Primary	Warming vial with heat gun to promote evaporation	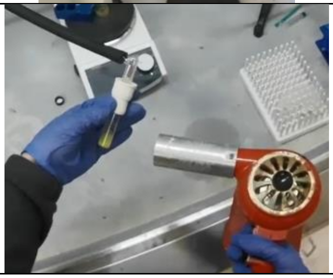	Warming item with hand so compound melts and is easier to handle<br>- Human body

E10	Human body	using the human body itself directly as the primary tool (e.g., using a hand to test the temperature of hot glass or tapping a finger against a flask to dislodge a stuck chemical)	Secondary	Warming item with hand so compound melts and is easier to handle	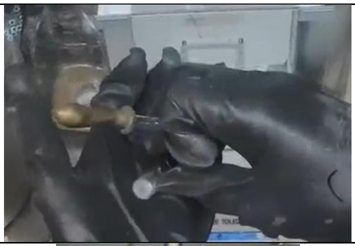	Washing hands at sink<br>- In-house utilities
E11	In-house utilities	using a room's non-scientific infrastructure (e.g., cabinets, sinks) – does not include glovebox, fume hood, oven, etc.	Primary	Washing hands at sink	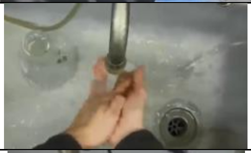	Putting NMR tubes in oven to dry them<br>- Oven
E12	Monkey bars & clamps	adjusting monkey bar, rod clamps, etc.	Primary	Adjusting clamps to hold flask to not fall over	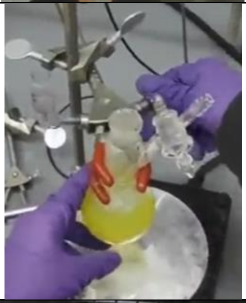	Adjusting thermometer to confirm reaction is at correct temperature<br>- Temperature bath
E13	Oven	using an oven	Primary	Putting NMR tubes in oven to dry them	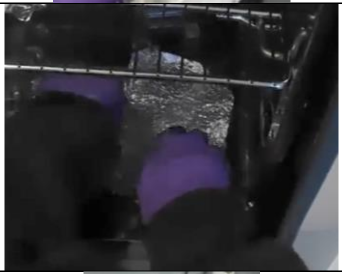	Putting on thermal gloves to prevent hand burn<br>- PPE
E14	Phone	operating a phone for scientific purposes (e.g., as a camera or a timer) – does not include recreational phone use	Primary	Referencing phone photo to decide which fractions to TLC	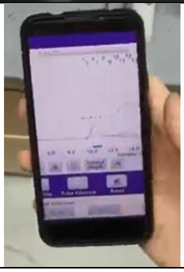	Removing gloves to protect non-lab materials<br>- PPE

E15	PPE	adjusting personal protective equipment (e.g., lab coat, safety glasses, gloves)	Primary	Putting new gloves on to protect hands	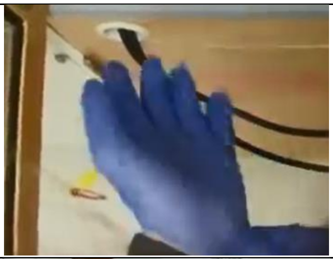	Lowering fume hood sash to conserve energy<br>- Fume hood
E16	Propane torch	operating a hand-held propane torch	Primary	Flaming flask to remove water	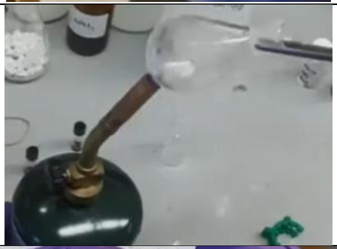	Putting on lab coat to work safely with flammable material<br>- PPE
E17	Records	using or creating records to communicate information to others or to future self	Primary	Labeling NMR cap to prevent sample mix-up	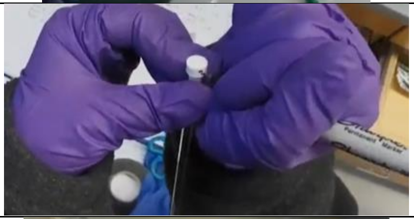	Calculating amounts to determine starting materials<br>- Calculator
E18	Rotatory evaporator	operating a rotatory evaporation machine and its built-in components (e.g., vacuum pump, water bath, bump trap) – does not include the attached flask/vial itself	Primary	Attaching collection flask to collect condensation	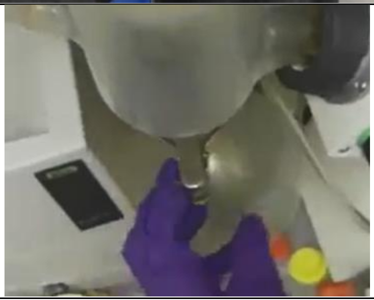	Holding magnet to keep stir bar from moving during rotatory evaporation<br>- Stir plate and bar magnet

E19	Schlenk line	operating a Schlenk line and its integral components (e.g., Dewar trap, needles in lines) – does not include the attached flask/vial itself	Primary	Switching line needle to enable a greater flow of inert gas	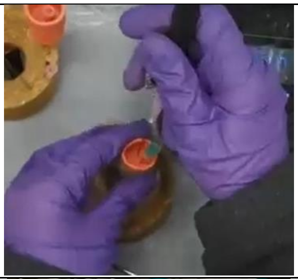		Preparing septum hookup to use Schlenk line<br>- Sundry items
E20	Solvent system	operating a solvent system (e.g., pump, stopcock) – does not include the associated transfer tools (e.g., syringe)	Primary	Draining solvent flask to get dry solvent	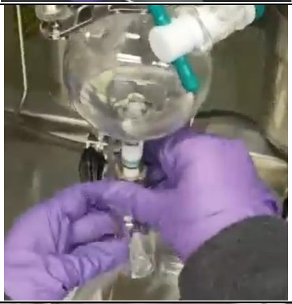		Drawing inert gas into needle to make headspace barrier<br>- Transfer tools – gas
E21	Sonicator	operating a sonicator	Primary	Operating sonicator to dislodge insoluble contaminant	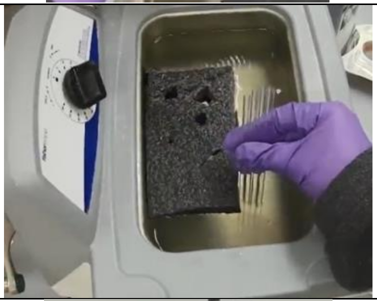		Swirling to dissolve solute<br>- Human body
E22	Standard glassware	adjusting glassware and its integral components (e.g., stopcocks in separatory funnel) – does not include attachments or other connected equipment (e.g., caps, Schlenk line adaptors, rotatory evaporator bump trap)	Secondary	Opening stopcock to drain emulsion onto vacuum filter funnel	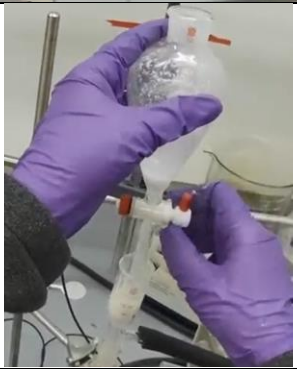		Capping flask to prevent a possible spill<br>- Sundry items

E23	Static gun	operating a static gun	Primary	Using static gun to stabilize balance measurement	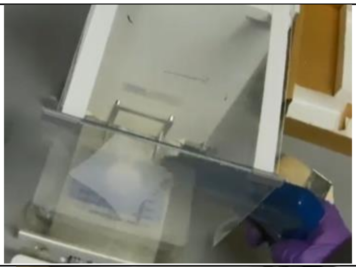	Fixing crooked balance tray to measure mass<br>- Balance
E24	Stir plate & bar magnet	operating a stir plate or another magnet to manipulate a stir bar	Primary	Holding magnet to keep stir bar from moving during rotatory evaporation	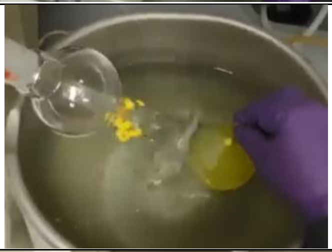	Rinsing stir bar to retrieve every bit of reaction mixture<br>- Transfer tools – liquid
E25	Sundry items	using (typically small) items not accounted for under other categories and often in conjunction with managing glassware (e.g., caps, tape)	Secondary	Capping flask to prevent a possible spill	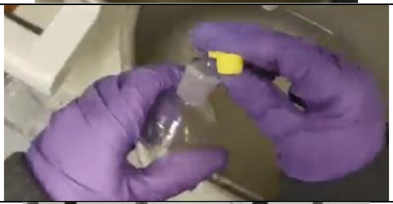	Opening stopcock to drain emulsion onto vacuum filter funnel<br>- Standard glassware
E26	Temperature bath	using a cold or hot bath and its integral accessories (e.g., thermometer, dry ice evaporation cover) – does not include when bath is part of another machine (e.g., rotatory evaporator)	Primary	Adjusting thermometer to confirm reaction is at correct temperature	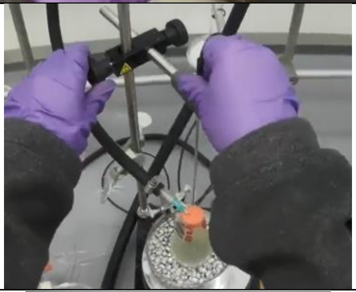	Touching bath and flask to doublecheck temperature<br>- Human body
E27	TLC chamber and stain	using a thin-layer chromatography elution / development chamber or stain solution	Primary	Placing plate in chamber to confirm presence of product in mixture	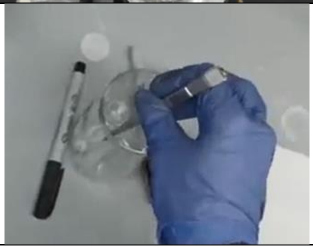	Using UV light to confirm spotting is heavy enough<br>- UV lamp

E28	Transfer tools – gas	using items to move a gas from one location to another (e.g., via syringe headspace)	Primary	Drawing inert gas into needle to make headspace barrier	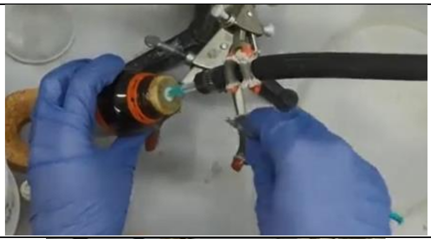	Drawing solvent into syringe to obtain reagent for reaction<br>- Transfer tools - liquid
E29	Transfer tools – liquid	using items to move a liquid from one location to another (e.g., via syringes, squirt bottles)	Primary	Adding solvent to try to dissolve solid	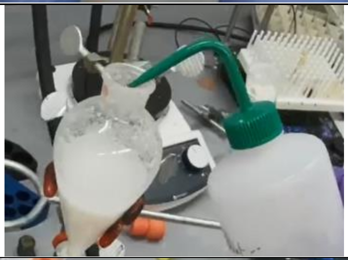	Drawing inert gas into needle to make headspace barrier<br>- Transfer tools – gas
E30	Transfer tools – solid	using items to move a solid from one location to another (e.g., via spatulas, weigh paper, wipes)	Primary	Scraping mass out of filter with spatula to collect product	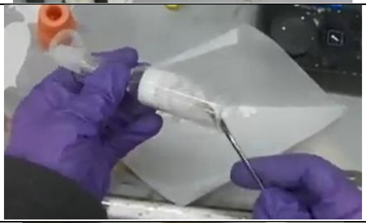	Adding solvent to try to dissolve solid<br>- Transfer tools – liquid
E31	UV lamp	using an ultraviolet lamp	Primary	Using UV light to confirm spotting is heavy enough	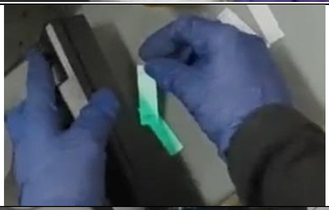	Dipping TLC plate in stain to reveal non-UV spots<br>- TLC chamber & stain
E32	Waste collector	using a waste collector to segregate hazardous waste	Primary	Collecting used silica in jar to dispose of safely	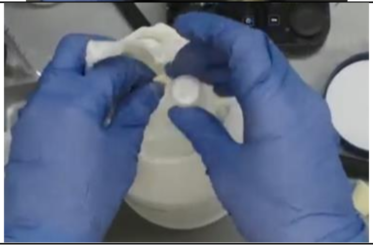	Preparing cleaning solution to clean TLC spotter<br>- Transfer tools – liquid

Table S2. Codebook for Technique.

#	Technique Code	Definition	Primary / Secondary	Applicable MA Example	Image of Applicable MA Example	NOT an Applicable MA Example
T1	Air-free mass transfer	preventing a chemical from being exposed to air while transferring from one location to another location	Primary	Drawing inert gas into needle to make headspace barrier	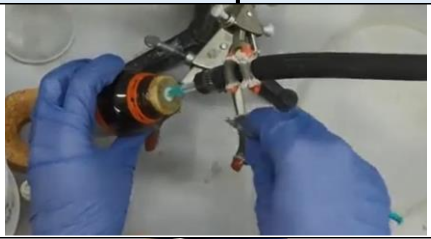	Scraping spatula on flask mouth to get residual to fall off<br>- Mass transfer – solid
T2	Airstream evaporation	using airflow to evaporate a chemical	Primary	Decreasing air flow to reduce loud annoying sound	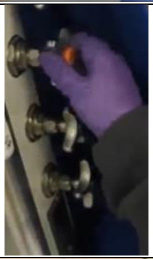	Switching line needle to enable a greater flow of inert gas<br>- Schlenk line operation
T3	Chemical drawing software usage	operating chemical drawing software to draw and obtain information about chemicals	Primary	Drawing in chemical drawing software to get molar mass for calculations	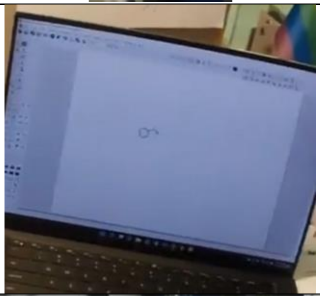	Calculating amounts to determine starting materials<br>- Notebook management
T4	Cleaning and waste disposal	cleaning up and disposing of hazardous waste	Primary	Collecting used silica in jar to dispose of safely	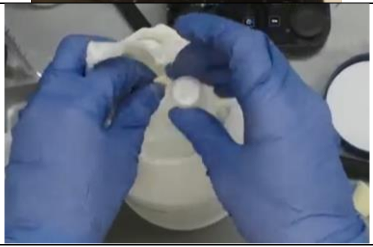	Attaching collection flask to collect condensation<br>- Rotatory evaporation

T5	Column chromatography	separating chemicals via column chromatography	Primary	Adjusting column conditions to finish up faster	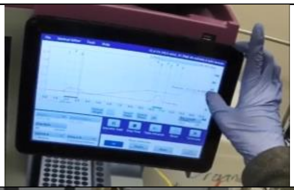	Pouring fraction into flask for collection<br>- Rotatory evaporation
T6	Dissolution	trying to dissolve a substance, especially one that is not dissolving easily – does not include when a reagent immediately dissolves after addition	Secondary	Adding solvent to try to dissolve solid	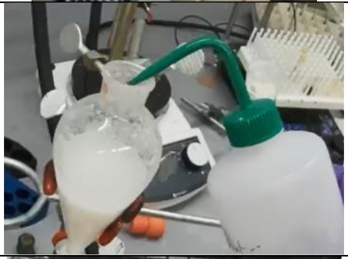	Operating sonicator to dislodge insoluble contaminant<br>- Sonication
T7	Glassware drying	removing trace water from glassware	Primary	Putting NMR tubes in oven to dry them	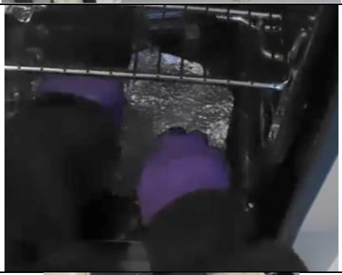	Waving plate to dry it after elution<br>- Thin-layer chromatography
T8	Glovebox operation	operating a glovebox	Primary	Evacuating glovebox chamber to ensure a tight door seal	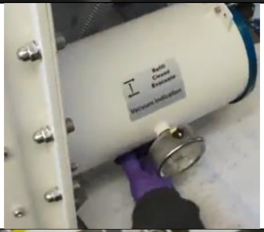	Adjusting clamps to hold flask to not fall over<br>- Laboratory and workspace management
T9	Inventory management	managing lab materials and stock supplies in accordance with accepted conventions	Secondary	Storing compound in fridge to prevent decomposition	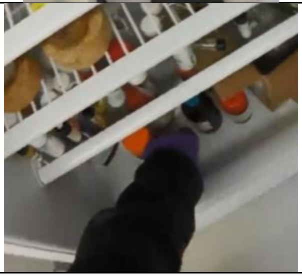	Putting NMR tubes in oven to dry them<br>- Glassware drying

T10	Laboratory and workspace management	managing lab infrastructure and general workspace in accordance with accepted conventions	Secondary	Adjusting clamps to hold flask to not fall over	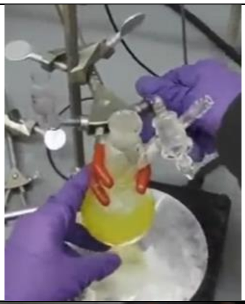	Evacuating glovebox chamber to ensure a tight door seal<br>- Glovebox operation
T11	Light-sensitive reaction management	preventing a chemical from being exposed to light	Primary	Covering flask in aluminum foil to protect light-sensitive reaction	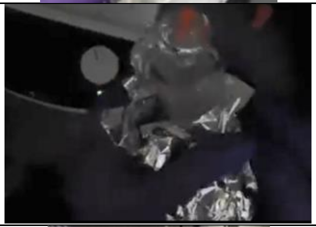	Capping flask to prevent a possible spill<br>- Inventory management
T12	Liquid-liquid extraction	separating chemicals via liquid-liquid extraction	Primary	Adding solvent to make a larger phase	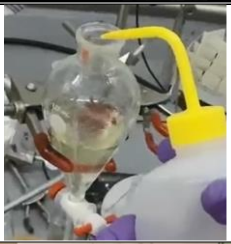	Opening stopcock to drain emulsion onto vacuum filter funnel<br>- Vacuum filtration
T13	Literature searching	finding information in the literature	Primary	Retrieving NMR data from an article to reference	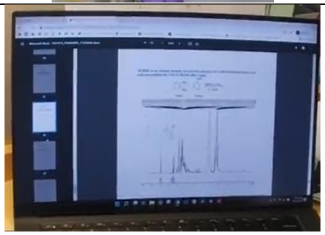	Drawing in chemical drawing software to get molar mass for calculations<br>- Chemical drawing software usage

T14	Mass measurement – liquid	measuring amount of a liquid	Primary	Pouring solvent into cylinder to measure amount for addition	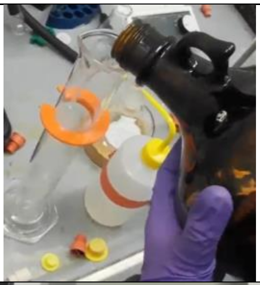	Pumping cleaning solvent through used syringe before discarding<br>- Cleaning and waste disposal
T15	Mass measurement – solid	measuring amount of a solid	Primary	Getting mass of weigh paper to see how much was not transferred	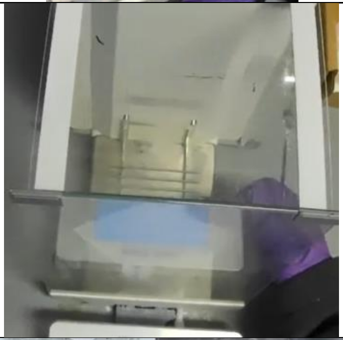	Scraping spatula on flask mouth to get residual to fall off<br>- Mass transfer – solid
T16	Mass transfer – liquid	moving a liquid from one location to another	Secondary	Depressing pipette bulb repeatedly to get every bit out	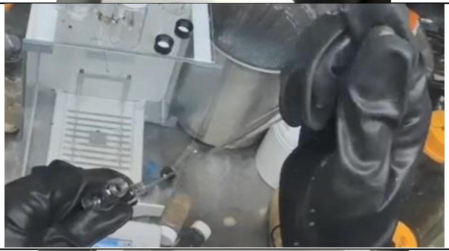	Draining solvent flask to get dry solvent<br>- Solvent system operation
T17	Mass transfer – solid	moving a solid from one location to another	Secondary	Scraping spatula on flask mouth to get residual to fall off	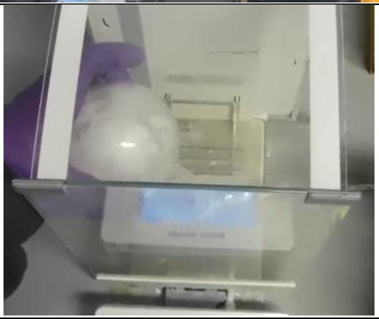	Collecting used silica in jar to dispose of safely<br>- Cleaning and waste disposal

T18	Mass transfer – phase change	manipulating the phase of a chemical to enable mass transfer in a different phase	Secondary	Warming item with hand so compound melts and is easier to handle	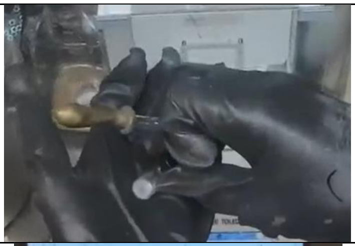	Warming vial with heat gun to promote evaporation<br>- Schlenk line operation
T19	NMR analysis	analyzing NMR data	Primary	Calibrating NMR integrals' proton count	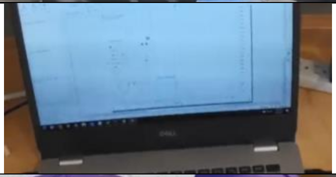	Retrieving NMR data from an article to reference<br>- Literature searching
T20	NMR sample preparation	making an NMR solution in an NMR tube	Primary	Labeling NMR cap to prevent sample mix-up	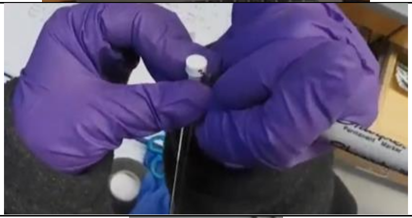	Depressing pipette bulb repeatedly to get every bit out<br>- Mass transfer – liquid
T21	Notebook management	setting up or recording data in experimental notebook	Primary	Calculating amounts to determine starting materials	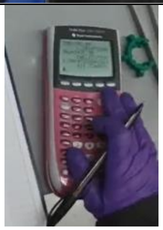	Drawing in chemical drawing software to get molar mass for calculations<br>- Chemical drawing software usage
T22	PPE management	managing own personal protective equipment	Primary	Putting new gloves on to protect hands	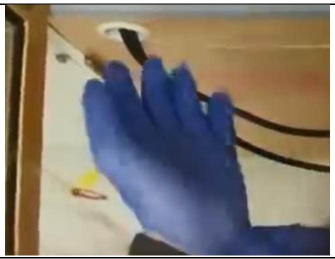	Decreasing air flow to reduce loud annoying sound<br>- Airstream evaporation

T23	Rotatory evaporation	operating a rotatory evaporator to evaporate a substance	Primary	Attaching collection flask to collect condensation	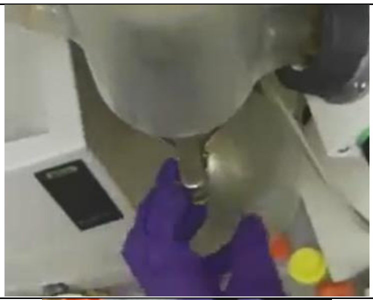	Adding silica to solution for later dry loading<br>- Column chromatograph y
T24	Schlenk line operation	operating a Schlenk line to apply vacuum or manage inert atmosphere	Primary	Switching line needle to enable a greater flow of inert gas	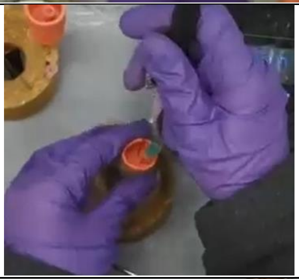	Drawing inert gas into needle to make headspace barrier<br>– Air-free mass transfer
T25	Solution drying and gravity filtration	removing trace water or filtering off solids from a solution	Primary	Adding sodium sulfate to dry solution	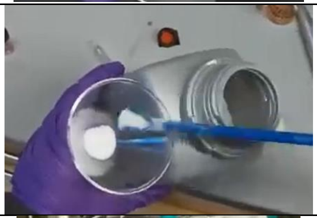	Opening stopcock to drain emulsion onto vacuum filter funnel<br>- Vacuum filtration
T26	Solvent system operation	operating a solvent system to collect dry solvent	Primary	Draining solvent flask to get dry solvent	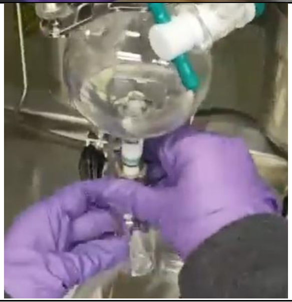	Depressing pipette bulb repeatedly to get every bit out<br>– Mass transfer – liquid

T27	Sonication	sonicating a mixture to improve homogeneity	Primary	Operating sonicator to dislodge insoluble contaminant	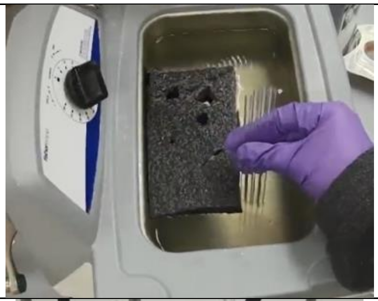	Swirling to dissolve solute<br>- Dissolution
T28	Temperature bath manipulation	monitoring, lowering, or raising the temperature of a reaction – does not include when bath is part of another machine (e.g., rotatory evaporator's warm water bath, Schlenk line vacuum pump's cold dewar)	Secondary	Adjusting thermometer to confirm reaction is at correct temperature	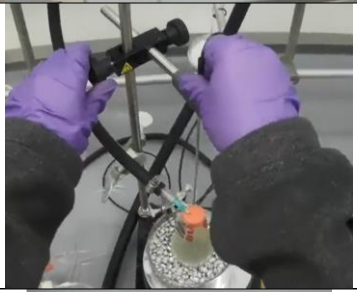	Checking water bath temperature to ensure it is good<br>– Rotatory evaporation
T29	Thin-layer chromatograp hy	separating chemicals via thin-layer chromatography	Primary	Placing plate in chamber to confirm presence of product in mixture	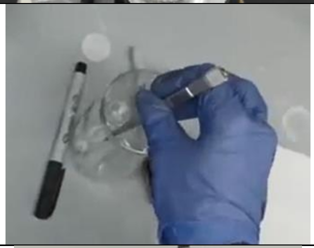	Using phone to take photo of TLC plate for notebook later<br>- Notebook management
T30	Vacuum filtration	applying a vacuum to separate solid and liquid components of a mixture	Primary	Opening stopcock to drain emulsion onto vacuum filter funnel	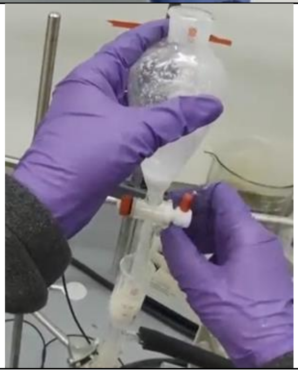	Applying vacuum to help pack column silica<br>- Column chromatograph y

Table S3. Codebook for Purpose.

#	Purpose Code	Definition	Primary / Secondary	Applicable MA Example	Image of Applicable MA Example	NOT an Applicable MA Example
P1	Analyze results	using a qualitative or quantitative test to try to determine whether synthesis or purification was successful	Primary	Retrieving NMR data from an article to reference	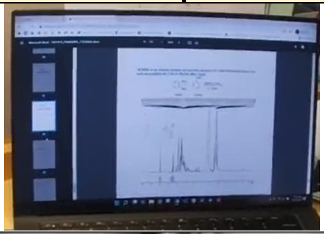	Labeling NMR cap to prevent sample mix-up<br>- Optimization – analyze results
P2	Avoid contamination	avoiding the potential for contamination to occur, including atmospheric contamination of oxygen, introduction of trace water to glassware, or other avenues for chemical contamination – does not include actively engaging in purification	Primary	Drawing inert gas into needle to make headspace barrier	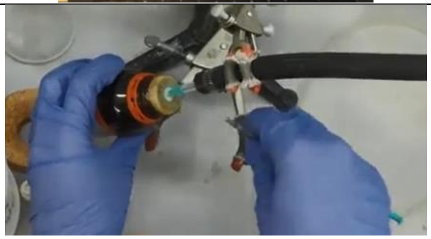	Applying air to dry out column so silica can be removed<br>- Optimization – avoid contamination
P3	Enable next step only	executing an action with no other Purpose than to operate specialized equipment in order to do the next step of a Technique	Secondary	Switching vacuum to nitrogen to release vial	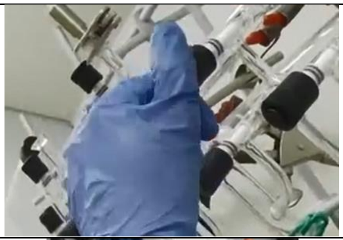	Writing in notebook to record work<br>- Enable repetition via records
P4	Enable repetition via measurement s	measuring materials so as to accurately record what was used or so as to completely use what was recorded as being used	Primary	Pouring solvent into cylinder to measure amount for addition	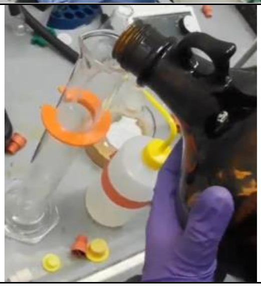	Writing on glove to remind self of the needed amount<br>– Optimization – enable repetition via measurements

P5	Enable repetition via records	enabling a procedure to be repeated in the future through record-keeping	Primary	Writing in notebook to record work	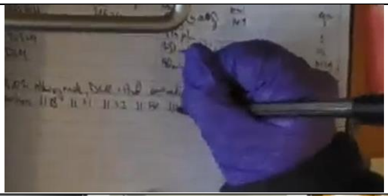	Labeling NMR cap to prevent sample mix-up<br>- Optimization – analyze results
P6	Maintain work relationships	maintaining positive work relationships by engaging in considerate behaviors for the benefit of labmates	Primary	Cleaning up spilled material under the balance tray	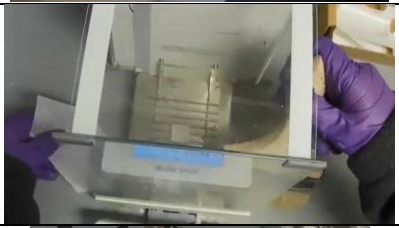	Decreasing air flow to reduce loud annoying sound<br>- Reduce personal annoyance
P7	Maximize product yield	maximizing the amount of product that can be collected or prevent loss of formed product	Primary	Adding solvent to try to dissolve solid	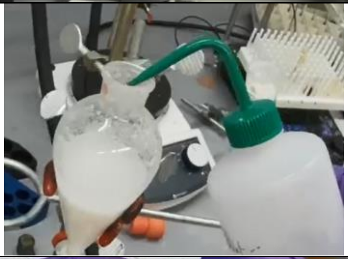	Feeling for a leak from the running column<br>- Optimization – maximize product yield
P8	*Optimization – analyze results*	doing something to facilitate analyzing results or to avoid needing to redo a task – does not include actions necessary on the surface to analyze results	Primary	Labeling NMR cap to prevent sample mix-up	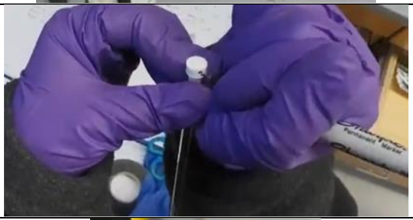	Retrieving NMR data from an article to reference<br>– Analyze results
P9	*Optimization – avoid contamination*	doing something to facilitate avoiding contamination or to avoid needing to redo a task – does not include actions necessary on the surface to avoid contamination	Primary	Applying air to dry out column so silica can be removed	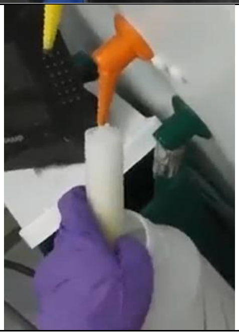	Drawing inert gas into needle to make headspace barrier<br>– Avoid contamination

P10	*Optimization – enable repetition via measurements*	doing something to facilitate measuring accurately or to avoid needing to redo a task – does not include actions necessary on the surface to measure accurately	Primary	Writing on glove to remind self of the needed amount	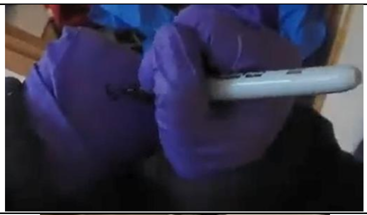	Pouring solvent into cylinder to measure amount for addition<br>– Enable repetition via measurements
P11	*Optimization – maximize product yield*	doing something to facilitate maximizing yield or to avoid needing to redo a task – does not include actions necessary on the surface to maximize product yield	Primary	Feeling for a leak from the running column	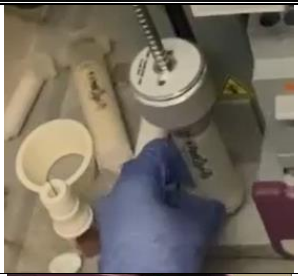	Adding solvent to try to dissolve solid<br>– Maximize product yield
P12	*Optimization – purify product*	doing something to facilitate purifying product or to avoid needing to redo a task – does not include actions necessary on the surface to purify product	Primary	Adjusting column conditions to finish up faster	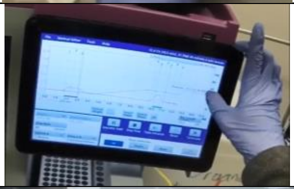	Applying vacuum to remove residual solvent<br>– Purify product
P13	*Optimization – synthesize product*	doing something to facilitate synthesizing product or to avoid needing to redo a task – does not include actions necessary on the surface to synthesize product	Primary	Warming item with hand so compound melts and is easier to handle	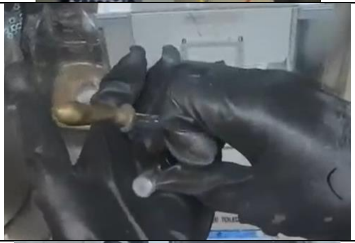	Calculating amounts to determine starting materials<br>– Optimization – synthesize product
P14	Preserve amount of supply	avoiding unnecessary loss of expendable materials (e.g., silica gel, starting material, argon atmosphere)	Primary	Trying to recover material stuck in pipette to not waste	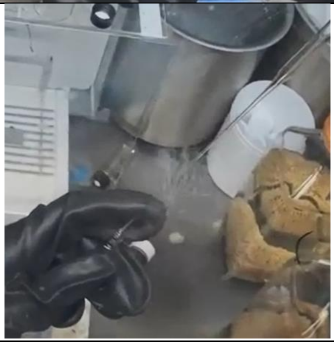	Storing compound in fridge to prevent decomposition<br>- Preserve product integrity

P15	Preserve product integrity	preventing product from degrading after formation	Primary	Storing compound in fridge to prevent decomposition	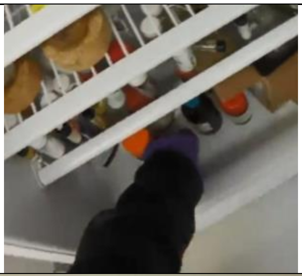	Applying vacuum to remove residual solvent<br>- Purify product
P16	Protect equipment	safeguarding the functionality of equipment	Primary	Hammering dry ice to make sizes fit for rotatory evaporator's condenser	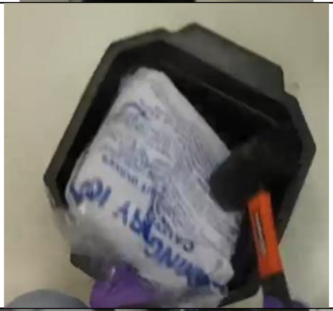	Storing compound in fridge to prevent decomposition<br>- Preserve product integrity
P17	Protect people	safeguarding the health of people	Primary	Collecting used silica in jar to dispose of safely	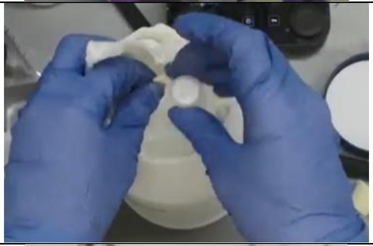	Cleaning up spilled material under the balance tray<br>- Maintain work relationships
P18	Purify product	separating desired product from contaminants – does not include avoidance of contamination in general	Primary	Applying vacuum to remove residual solvent	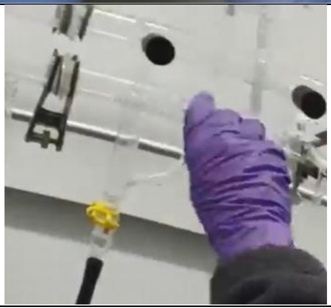	Adjusting column conditions to finish up faster<br>- Optimization – purify product

P19	Reduce personal annoyance	managing a personal source of vexation to be less annoying	Primary	Decreasing air flow to reduce loud annoying sound	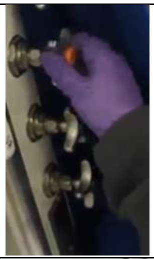	Cleaning up spilled material under the balance tray<br>- Maintain work relationships
P20	Synthesize product	initiating a chemical change to try to create a desired product	Primary	Calculating amounts to determine starting materials	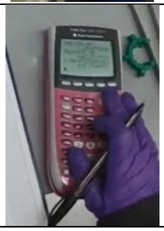	Warming item with hand so compound melts and is easier to handle<br>- Synthesize product

Table S4. Text Analysis of Video Clip with Example Coding.

Meaningful Action	ETP Coding	Rationale
using tweezers to pick up TLC plate to avoid contamination	E: transfer tools – solid<br>T: thin-layer chromatography<br>P: optimization - avoid contamination	A transfer tool (tweezers) is being used to manipulate a TLC plate. This process is the beginning of preparing the materials for thin-layer chromatography. If the tweezers weren't used, the participant would find it harder to keep the TLC plate clean and free of contaminants from their gloves.
marking plate as contaminated to avoid using	E: records<br>T: thin-layer chromatography<br>P: optimization - analyze results	A TLC plate is being marked as contaminated. If the plate wasn't marked, the participant might make a mistake and run TLC using the contaminated plate, meaning they would have to then redo the TLC when they realize it was contaminated upon analysis.
marking a TLC plate to keep track of spots	E: records<br>T: thin-layer chromatography<br>P: analyze results	If the spots' loading locations weren't marked, the participant wouldn't be able to analyze the results accurately.
spotting a TLC plate with standard to determine compound presence	E: transfer tools – liquid<br>T: thin-layer chromatography<br>P: analyze results	If the plate wasn't spotted with standard, the participant wouldn't be able to analyze the results accurately.
spotting a TLC spotter with clean solvent to remove contamination	E: transfer tools – liquid<br>T: cleaning and waste disposal<br>P: avoid contamination	The TLC spotter is being cleaned. If the spotter wasn't cleaned out, future samples would be contaminated the next time the spotter is used.
re-spotting a TLC spotter with solvent to ensure de-contamination	E: transfer tools – liquid<br>T: cleaning and waste disposal<br>P: optimization - avoid contamination	If the spotter wasn't cleaned multiple times, the participant wouldn't be confident about whether it is / isn't actually clean.
marking a TLC plate to keep track of conditions	E: records<br>T: thin-layer chromatography<br>P: enable repetition via records	If the plate wasn't marked with the conditions, the participant wouldn't have a record of the conditions that they should use again in the future.
spotting a TLC plate with mixture to determine composition	E: transfer tools – liquid<br>T: thin-layer chromatography<br>P: analyze results	If the plate wasn't spotted with the crude mixture, the participant wouldn't be able to analyze the results.
waving plate to dry it before elution	E: human body<br>T: thin-layer chromatography<br>P: optimization - analyze results	The participant is using their own body as a tool to provide airflow and encourage the spots to dry quickly. If the plate wasn't waved around, the participant would have to wait longer before beginning elution.

What was not coded:

- Waiting for the spots to dry
    - This was an unseen cognitive decision. Since it did not physically manifest in a specific psychomotor action within the clip, it did not meet the criteria for coding.
- Rubbing gloved fingers against each other
    - This did not readily offer clear meaning as a separate meaningful action, and so it did not meet the criteria for coding.
- Emptying the spotter of remaining solvent after picking it up from the vial of cleaning solvent
    - Emptying the spotter is a natural and necessary next-step for using the spotter, and so it did not meet the criteria for coding.
- Moving head in closer to view the spot sizes more closely while spotting
    - The action was not directly viewable on the screen (only implied), and so it did not meet the criteria for coding.

Figure S1. Temporal Analysis of Intra- & Inter-Coder Reliability for MA Coding

This is an sample 10-minute video clip's temporal analysis (bottom → top). The clip was coded by the author twice and by a participant once for qualitative intra- & inter-coder reliability in MA coding. Items which aren't physical (i.e., "considering") and therefore couldn't be coded by the author are crossed out.

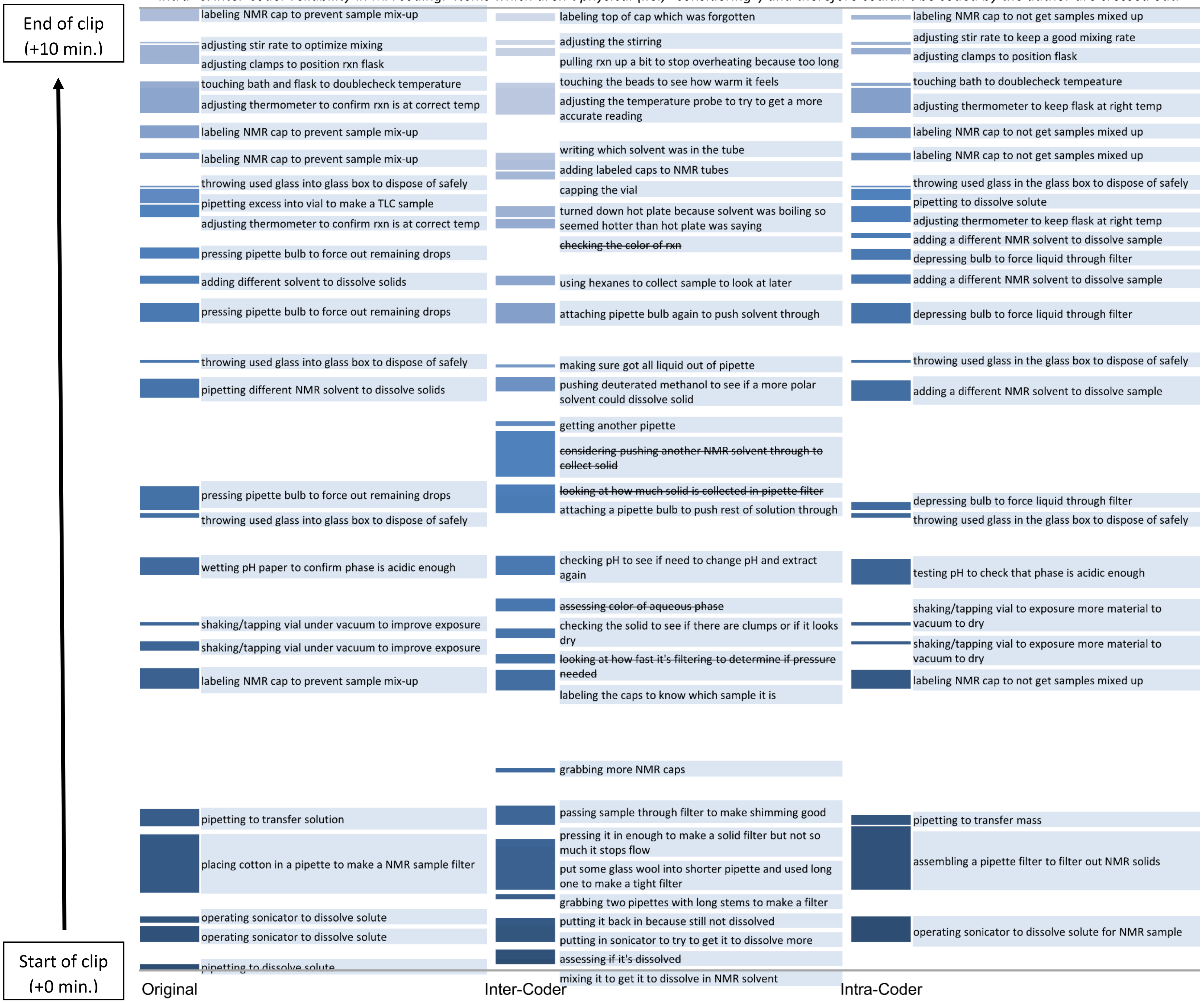

Supplement: Supplementary file 1 — ed2c00809_si_001.pdf [file ed2c00809_si_001.pdf]
