# Supplementary material for: A Day in the Life: Characterization of Doctoral Bench Research in Synthetic Chemistry Using Phenomenological Case Studies
Source: J Chem Educ. 2022 Dec 28;100(2):442–58. doi: 10.1021/acs.jchemed.2c00809 (PMC9933917; doi:10.1021/acs.jchemed.2c00809)

# Supporting Information: Cluster Analysis

## *A Day in the Life: Characterization of Doctoral Bench Research in Synthetic Chemistry using Phenomenological Case Studies*

Elizabeth W. Kelley*

Chemistry Department, University of Chicago, 5735 S Ellis Ave, Chicago, IL, 60637, USA

*ewkelley@uchicago.edu

**CONTENTS**

- CrossTabs count data for Equipment, Technique, and Purpose codes p. 2-4
  - The count data was used to conduct the cluster analysis.
- Cluster silhouette values and cluster sizes p. 5
  - Silhouette values demonstrate the fit of the cluster analysis for an 8-cluster model.
- Cluster count composition graphs p. 6-9
  - These graphs illustrate the codes which clustered into each cluster and the proportion they represent of their overall code categories.

# Table S5.  CrossTabs between Equipment and Purpose.

| | PURPOOSE | Analyze results | Avoid contamination | Enable next step only | Enable repetition via measurements | Enable repetition via records | Maintain work relationships | Maximize product yield | Optimization - analyze results | Optimization - avoid contamination | Optimization - enable repetition via measurements | Optimization - maximize product yield | Optimization - purify product | Optimization - synthesize product | Preserve amount of supply | Preserve product integrity | Protect equipment | Protect people | Purify product | Reduce personal annoyance | Synthesize product | TOTAL |
|---|---|---|---|---|---|---|---|---|---|---|---|---|---|---|---|---|---|---|---|---|---|---|
| EQUIPMENT | REF # | P1 | P2 | P3 | P4 | P5 | P6 | P7 | P8 | P9 | P10 | P11 | P12 | P13 | P14 | P15 | P16 | P17 | P18 | P19 | P20 | |
| Automated column | E1 | | | | | 4 | 1 | | | | | 2 | 13 | | | | | | 15 | | | 35 |
| Balance | E2 | | | | 65 | | | | | | 4 | | | | | | | | | 2 | | 71 |
| Calculator | E3 | 1 | | | 1 | 16 | | | | | | | | | | | | | | | 17 | 35 |
| Column | E4 | | 2 | | | | | | | | | 1 | | | | | | | 16 | | | 19 |
| Computer | E5 | 116 | | | | | | | | | | | 1 | | | | | | | | 1 | 118 |
| Fridge | E6 | | | | | | | | | | | | | | | 1 | | | | | | 1 |
| Fume hood | E7 | | 18 | 1 | | | | 1 | | 5 | | 1 | 6 | | | | 50 | 1 | 5 | 74 | | 162 |
| Glovebox | E8 | | 7 | 18 | | | | | | | | | | | | | 2 | | | | | 27 |
| Heat gun | E9 | 4 | 3 | | | | | | | | | | 5 | | | | | | | | | 12 |
| Human body | E10 | 4 | 64 | | | | | 70 | 6 | 23 | 1 | 21 | 37 | 18 | | | 2 | | 55 | | | 301 |
| In-house utilities | E11 | | 5 | | | | 26 | | | | | | | | | | | 2 | | | | 33 |
| Monkey bars & clamps | E12 | | | | | | | | | 1 | | 1 | 11 | | | | 3 | | | | 32 | 48 |
| Oven | E13 | | 6 | | | | | | | | | | | | | | | | | | | 6 |
| Phone | E14 | | | | | 10 | | | 4 | | | | | 1 | | | | | | | 1 | 16 |
| PPE | E15 | | | | | | | | | | | | | | | | | 75 | | 6 | | 81 |
| Propane torch | E16 | | 3 | | | | | | | | | | | | | | | | | | | 3 |
| Records | E17 | 33 | | | | 49 | 8 | | 9 | | 2 | | | 2 | | 10 | | 1 | | 2 | 1 | 117 |
| Rotatory evaporator | E18 | | 8 | | | | | | | | | 61 | 119 | | | | 2 | | 8 | | | 198 |
| Schlenk line | E19 | | 12 | 5 | | | | | | | | 2 | 9 | | 2 | | 6 | | 9 | | | 45 |
| Solvent system | E20 | | 1 | | | | | 1 | | | | | | | 1 | | | | | | | 3 |
| Sonicator | E21 | | 2 | | | | | | | | | 10 | | | | | | | | | | 12 |
| Standard glassware | E22 | | 1 | 1 | | | 4 | | 1 | 1 | | 66 | 6 | 19 | 1 | | | | 64 | | | 164 |
| Static gun | E23 | | | | | | | | | | 8 | | | 1 | | | | | | | | 9 |
| Stir plate & bar magnet | E24 | | | | | | | | | | | | | 6 | | | 5 | | | | | 11 |
| Sundry items | E25 | 1 | 21 | 1 | | | 3 | 2 | 32 | 2 | | 14 | 11 | 4 | 12 | 1 | 5 | 5 | | 4 | 9 | 127 |
| Temperature bath | E26 | | | | | | | | | | | | | 4 | 1 | | | | | | 6 | 11 |
| TLC chamber & stain | E27 | 16 | | | | | | | 2 | | | | | | | | | | | | | 18 |
| Transfer tools - gas | E28 | | 13 | | 1 | | | | | | | | | | | | | | | | | 14 |
| Transfer tools - liquid | E29 | 52 | 156 | 12 | 10 | | 1 | 83 | 15 | | | 1 | 8 | | 3 | | 8 | 2 | 63 | 1 | 55 | 470 |
| Transfer tools - solid | E30 | 15 | 34 | | 10 | | 2 | 2 | 3 | 40 | 15 | 7 | | 11 | 1 | | 11 | 7 | 14 | 9 | 33 | 214 |
| UV lamp | E31 | 16 | | | | | | | 9 | | | | | | | | | | | | | 25 |
| Waste collector | E32 | | 9 | | | | | | | | | | | | | | | 32 | | | | 41 |
| TOTAL | | 258 | 365 | 38 | 87 | 79 | 45 | 159 | 81 | 72 | 30 | 187 | 226 | 66 | 21 | 12 | 94 | 125 | 249 | 98 | 155 | 2447 |

## Table S6. CrossTabs between Equipment and Technique.

| | TECHNIQUE | Air-free mass transfer | Airstream evaporation | Chemical drawing software usage | Cleaning & waste disposal | Column chromatography | Dissolution | Glassware drying | Glovebox operation | Inventory management | Laboratory & workspace management | Light-sensitive reaction management | Liquid-liquid extraction | Literature searching | Mass measurement - liquid | Mass measurement - solid | Mass transfer - liquid | Mass transfer - phase change | Mass transfer - solid | NMR analysis | NMR sample preparation | Notebook management | PPE management | Rotatory evaporation | Schlenk line operation | Solution drying & gravity filtration | Solvent system operation | Sonication | Temperature bath manipulation | Thin-layer chromatography | Vacuum filtration | TOTAL |
|---|---|---|---|---|---|---|---|---|---|---|---|---|---|---|---|---|---|---|---|---|---|---|---|---|---|---|---|---|---|---|---|---|
| EQUIPMENT | REF # | T1 | T2 | T3 | T4 | T5 | T6 | T7 | T8 | T9 | T10 | T11 | T12 | T13 | T14 | T15 | T16 | T17 | T18 | T19 | T20 | T21 | T22 | T23 | T24 | T25 | T26 | T27 | T28 | T29 | T30 | |
| Automated column | E1 | | | | 1 | 34 | | | | | | | | | | | | | | | | | | | | | | | | | | 35 |
| Balance | E2 | | | | | | | | | | | | | | 2 | 69 | | | | | | | | | | | | | | | | 71 |
| Calculator | E3 | | | | | | | | | | | | | | | | | | | | | 34 | | | | | | | | 1 | | 35 |
| Column | E4 | | | | 2 | 17 | | | | | | | | | | | | | | | | | | | | | | | | | | 19 |
| Computer | E5 | | | 1 | | 1 | | | | | | | | 3 | | | | | | 113 | | | | | | | | | | | | 118 |
| Fridge | E6 | | | | | | | | | 1 | | | | | | | | | | | | | | | | | | | | | | 1 |
| Fume hood | E7 | | 7 | | 23 | 5 | | | | 1 | 123 | 1 | | | | | | | | | | | | | 1 | | | | | | 1 | 162 |
| Glovebox | E8 | | | | | | | 1 | 26 | | | | | | | | | | | | | | | | | | | | | | | 27 |
| Heat gun | E9 | | | | 3 | | | | | | | | | | | | | | | | | | | | 5 | | | | | 4 | | 12 |
| Human body | E10 | 1 | | | 23 | 17 | 35 | 3 | | 3 | 2 | | 83 | | | 9 | 1 | 4 | 33 | | 1 | | | 10 | 34 | 1 | | | 3 | 31 | 7 | 301 |
| In-house utilities | E11 | | | | 3 | | | | | | 1 | | | | | | | | | | | | 3 | 26 | | | | | | | | 33 |
| Monkey bars & clamps | E12 | 9 | | | | | | | | | 39 | | | | | | | | | | | | | | | | | | | | | 48 |
| Oven | E13 | | | | | | | 6 | | | | | | | | | | | | | | | | | | | | | | | | 6 |
| Phone | E14 | | | | | | | | | | | | | | | | | | | | | 12 | | | | | | | | 4 | | 16 |
| PPE | E15 | | | | | | | | | | 3 | | | | | | | | | | | | 78 | | | | | | | | | 81 |
| Propane torch | E16 | | | | | | | 3 | | | | | | | | | | | | | | | | | | | | | | | | 3 |
| Records | E17 | | | | | | | | 7 | 12 | | | | | | 2 | | | | | 8 | 50 | | | | | 3 | | | 35 | | 117 |
| Rotatory evaporator | E18 | | | | | | | | | | | | | | | | | | | | | | | 198 | | | | | | | | 198 |
| Schlenk line | E19 | | | | | | | | | | | | | | | | | | | | | | | | 45 | | | | | | | 45 |
| Solvent system | E20 | | | | | | | | | | | | | | | | | | | | | | | | | | 3 | | | | | 3 |
| Sonicator | E21 | | | | | | | | | | | | | | | | | | | | | | | | | | | 12 | | | | 12 |
| Standard glassware | E22 | | | | | 15 | | | | 1 | 1 | | 109 | | | | 7 | 2 | 9 | | 1 | | | 2 | 4 | 6 | | | | | 7 | 164 |
| Static gun | E23 | | | | | | | | | | | | | | | 8 | | | 1 | | | | | | | | | | | | | 9 |
| Stir plate & bar magnet | E24 | | | | 3 | | 5 | | | | 1 | | | | | | | | | | | | | 2 | | | | | | | | 11 |
| Sundry items | E25 | 1 | | | 6 | | | | 6 | 14 | 25 | 2 | 6 | | | | | | 9 | | | | 2 | 18 | 8 | | | | | 30 | | 127 |
| Temperature bath | E26 | | | | | | | | | | | | | | | | | 2 | | | | | | | | | | | 9 | | | 11 |
| TLC chamber & stain | E27 | | | | | | | | | | | | | | | | | | | | | | | | | | | | | 18 | | 18 |
| Transfer tools - gas | E28 | 13 | | | | | | | | | | | | | 1 | | | | | | | | | | | | | | | | | 14 |
| Transfer tools - liquid | E29 | 15 | | | 154 | 14 | 7 | | | 14 | | | 65 | | 26 | | 56 | | 9 | | 18 | | | 33 | 4 | 1 | 1 | | 1 | 47 | 5 | 470 |
| Transfer tools - solid | E30 | | | | 33 | 15 | 2 | 2 | 9 | | | | | | | 4 | | | 66 | | | | | 16 | 2 | 3 | | | 2 | 57 | 3 | 214 |
| UV lamp | E31 | | | | | | | | | | | | | | | | | | | | | | | | | | | | | 25 | | 25 |
| Waste collector | E32 | | | | 41 | | | | | | | | | | | | | | | | | | | | | | | | | | | 41 |
| TOTAL | | 39 | 7 | 1 | 292 | 118 | 49 | 15 | 48 | 46 | 195 | 3 | 263 | 3 | 29 | 92 | 64 | 8 | 127 | 113 | 28 | 96 | 83 | 305 | 103 | 11 | 7 | 12 | 15 | 252 | 23 | 2447 |

*Numbers are squished vertically to fit on the page (e.g., 1/3 is 13).*

## Table S7. CrossTabs between Technique and Purpose.

| | PURPOSE | Analyze results | Avoid contamination | Enable next step only | Enable repetition via measurements | Enable repetition via records | Maintain work relationships | Maximize product yield | Optimization - analyze results | Optimization - avoid contamination | Optimization - enable repetition via measurements | Optimization - maximize product yield | Optimization - purify product | Optimization - synthesize product | Preserve amount of supply | Preserve product integrity | Protect equipment | Protect people | Purify product | Reduce personal annoyance | Synthesize product | TOTAL |
|---|---|---|---|---|---|---|---|---|---|---|---|---|---|---|---|---|---|---|---|---|---|---|
| **TECHNIQUE** | **REF #** | **P1** | **P2** | **P3** | **P4** | **P5** | **P6** | **P7** | **P8** | **P9** | **P10** | **P11** | **P12** | **P13** | **P14** | **P15** | **P16** | **P17** | **P18** | **P19** | **P20** | |
| **Air-free mass transfer** | **T1** | | 14 | | | | | | | | | | | | | | | 1 | | | 24 | **39** |
| **Airstream evaporation** | **T2** | | | | | | | | | | | 1 | 1 | | | | | | 4 | 1 | | **7** |
| **Chemical drawing software usage** | **T3** | | | | | | | | | | | | | | | | | | | | 1 | **1** |
| **Cleaning & waste disposal** | **T4** | | 242 | | | | 3 | | 1 | 6 | | | | | | | 3 | 36 | | 1 | | **292** |
| **Column chromatography** | **T5** | | | | | 4 | 2 | 6 | | 1 | | 10 | 29 | | | | | | 66 | | | **118** |
| **Dissolution** | **T6** | | | | | | | 41 | | | | 2 | | 5 | | | | | | | 1 | **49** |
| **Glassware drying** | **T7** | | 10 | | | | | | | | | | | 3 | | | | 2 | | | | **15** |
| **Glovebox operation** | **T8** | | 12 | 18 | | | 5 | | | | | | | | | | 2 | | | 11 | | **48** |
| **Inventory management** | **T9** | | 6 | 12 | | | 4 | | | | | 5 | | | 1 | 12 | | 2 | | 2 | 2 | **46** |
| **Laboratory & workspace management** | **T10** | | 4 | 2 | | | | | 2 | 3 | | 1 | 22 | 5 | 1 | | 53 | 2 | | 77 | 23 | **195** |
| **Light-sensitive reaction management** | **T11** | | | | | | | 2 | | | | 1 | | | | | | | | | | **3** |
| **Liquid-liquid extraction** | **T12** | 1 | 19 | | | | 2 | 24 | | | | 79 | 1 | | | | 4 | | 133 | | | **263** |
| **Literature searching** | **T13** | 3 | | | | | | | | | | | | | | | | | | | | **3** |
| **Mass measurement - liquid** | **T14** | | | | 13 | | | 9 | | | | | | | | | | | | | 7 | **29** |
| **Mass measurement - solid** | **T15** | | 9 | | 67 | | | | | | 14 | | | | | | | | | 2 | | **92** |
| **Mass transfer - liquid** | **T16** | 4 | | | | | | 26 | | | 1 | 1 | | 6 | 2 | | | | 3 | | 21 | **64** |
| **Mass transfer - phase change** | **T17** | | | | | | | | | | | | | 8 | | | | | | | | **8** |
| **Mass transfer - solid** | **T18** | | 6 | | 6 | | | 23 | 1 | | 15 | | | 26 | 1 | | | | 1 | | 48 | **127** |
| **NMR analysis** | **T19** | 113 | | | | | | | | | | | | | | | | | | | | **113** |
| **NMR sample preparation** | **T20** | 17 | 1 | | | | | | 9 | | | | | | | | | | 1 | | | **28** |
| **Notebook management** | **T21** | 1 | | | | 74 | | | | | | | | 3 | | | | | | | 18 | **96** |
| **PPE management** | **T22** | | 3 | | | | | | | | | | | | | | | 77 | | 3 | | **83** |
| **Rotatory evaporation** | **T23** | | 12 | | | | 26 | 15 | | 2 | | 71 | 129 | | 10 | | 22 | 5 | 13 | | | **305** |
| **Schlenk line operation** | **T24** | | 21 | 5 | | | | | | 1 | | 3 | 44 | 6 | 2 | | 10 | | 10 | 1 | | **103** |
| **Solution drying & gravity filtration** | **T25** | | | | | | | 2 | | | | | | | | | | | 9 | | | **11** |
| **Solvent system operation** | **T26** | | 1 | | | | 3 | 1 | | | | | | | 1 | | | | | | 1 | **7** |
| **Sonication** | **T27** | | 2 | | | | | | | | | 10 | | | | | | | | | | **12** |
| **Temperature bath manipulation** | **T28** | | | | | | | | | | | 1 | | 4 | 1 | | | | | | 9 | **15** |
| **Thin-layer chromatography** | **T29** | 119 | 2 | | 1 | 1 | | | 68 | 59 | | | | | 2 | | | | | | | **252** |
| **Vacuum filtration** | **T30** | | 1 | 1 | | | | 10 | | | | 2 | | | | | | | 9 | | | **23** |
| **TOTAL** | | **258** | **365** | **38** | **87** | **79** | **45** | **159** | **81** | **72** | **30** | **187** | **226** | **66** | **21** | **12** | **94** | **125** | **249** | **98** | **155** | **2447** |

**Figure S2. Cluster Sizes for an 8-Cluster Model.**

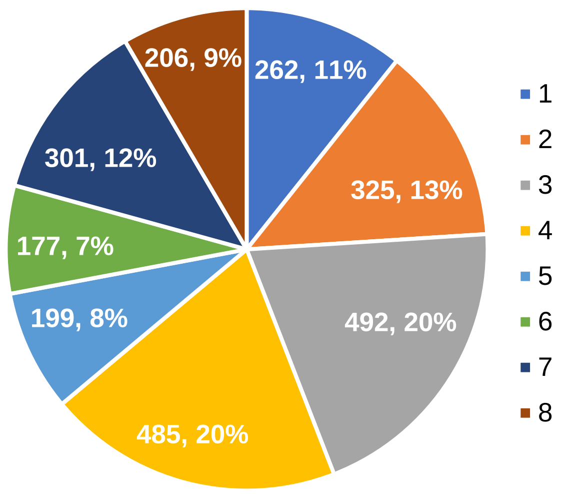


**Figure S3. Silhouette Values for an 8-Cluster Model.**

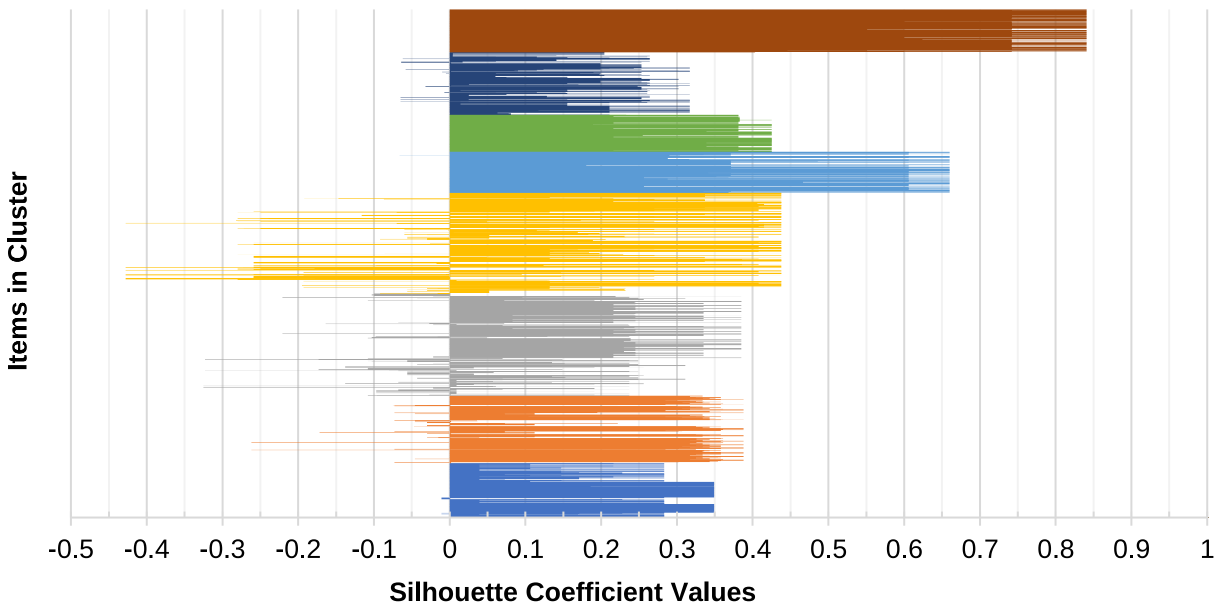


*Average k = .2670 (8-cluster local maximum k after testing up to 10 clusters)*

**Figure S4. Summary of Cluster 1: Analyzing NMRs and Preparing Chemicals.**

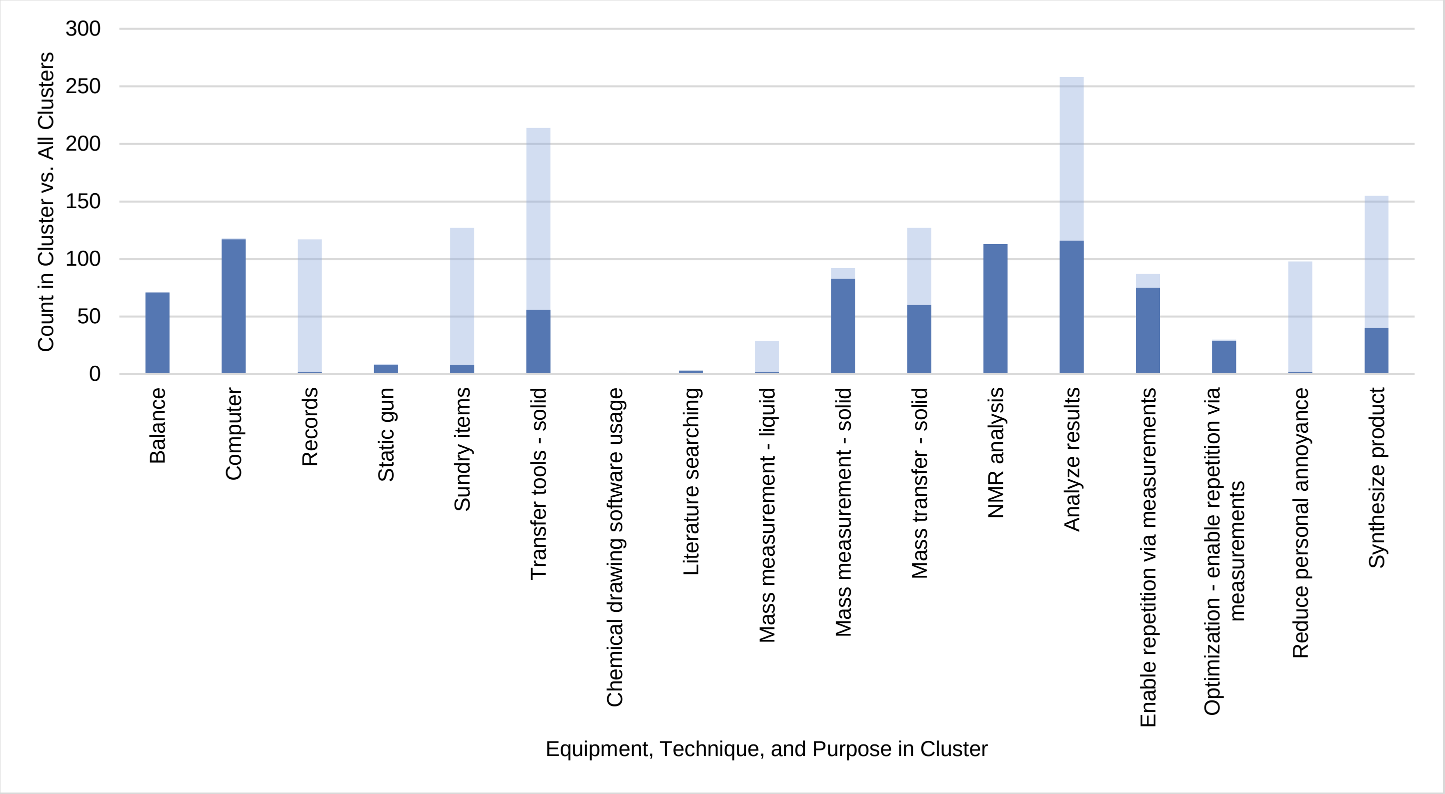


**Figure S5. Summary of Cluster 2: Making NMR Samples and Running TLCs.**

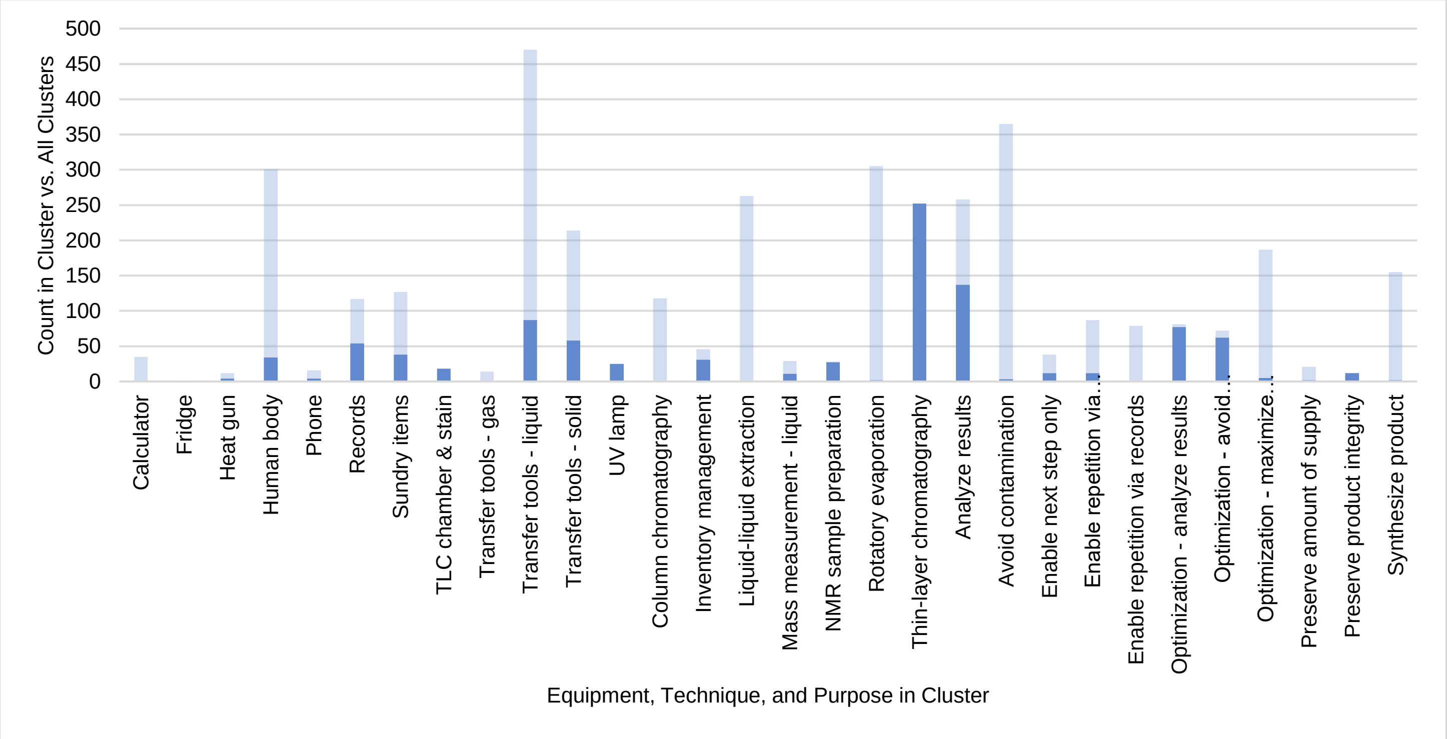

**Figure S6. Summary of Cluster 3: Purifying Crude Mixtures.**

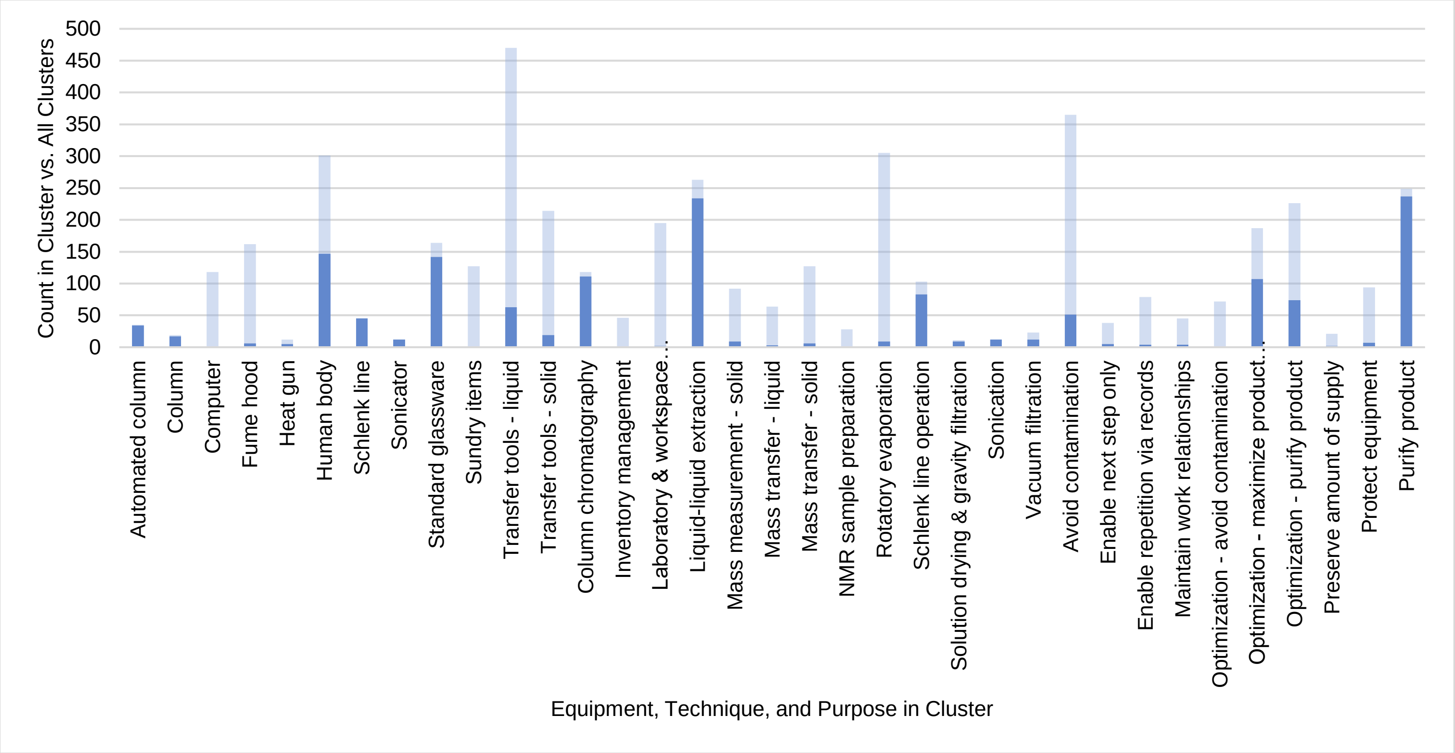


**Figure S7. Summary of Cluster 4: Using Scientific Equipment and Room Infrastructure.**

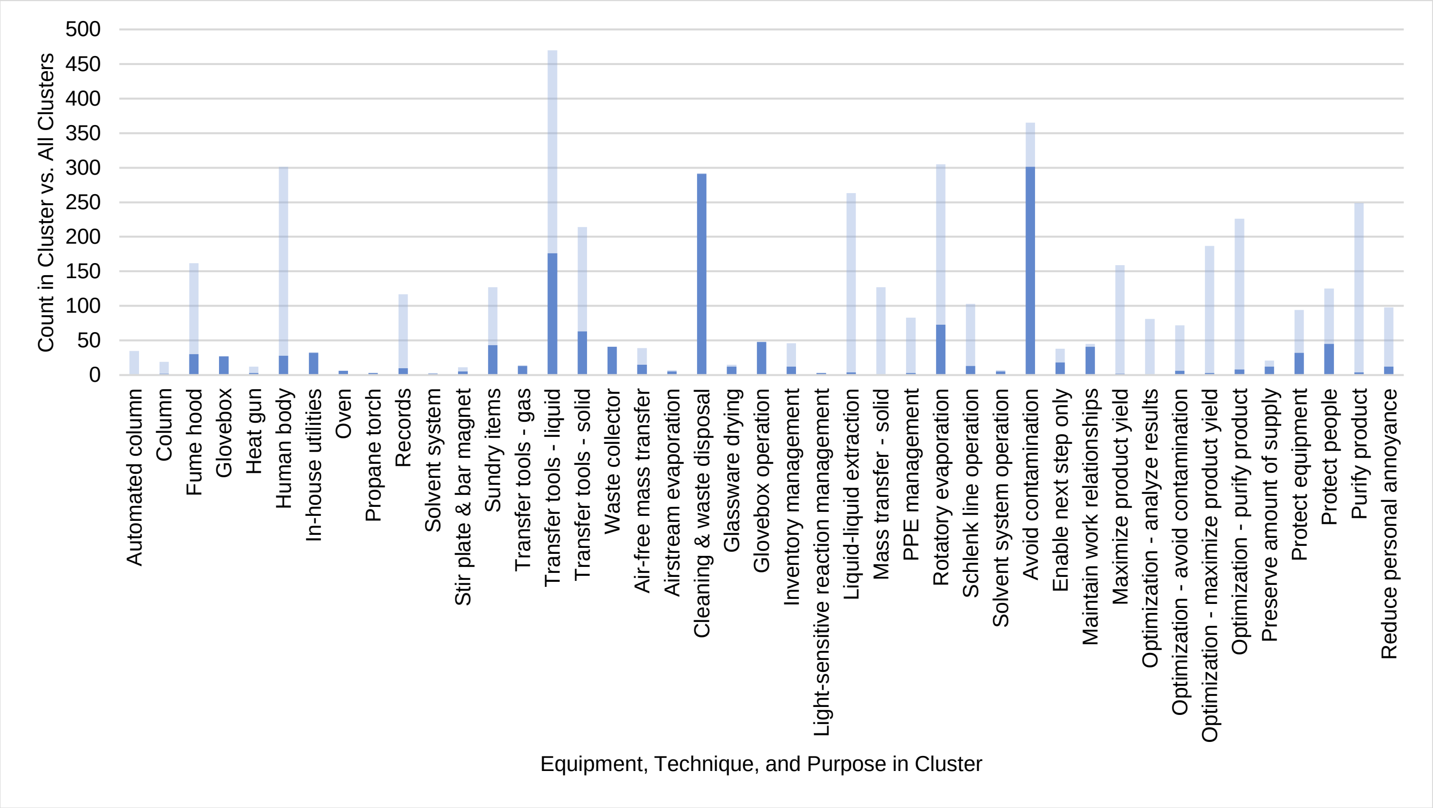

**Figure S8.  Summary of Cluster 5: Working in a Fume Hood.**

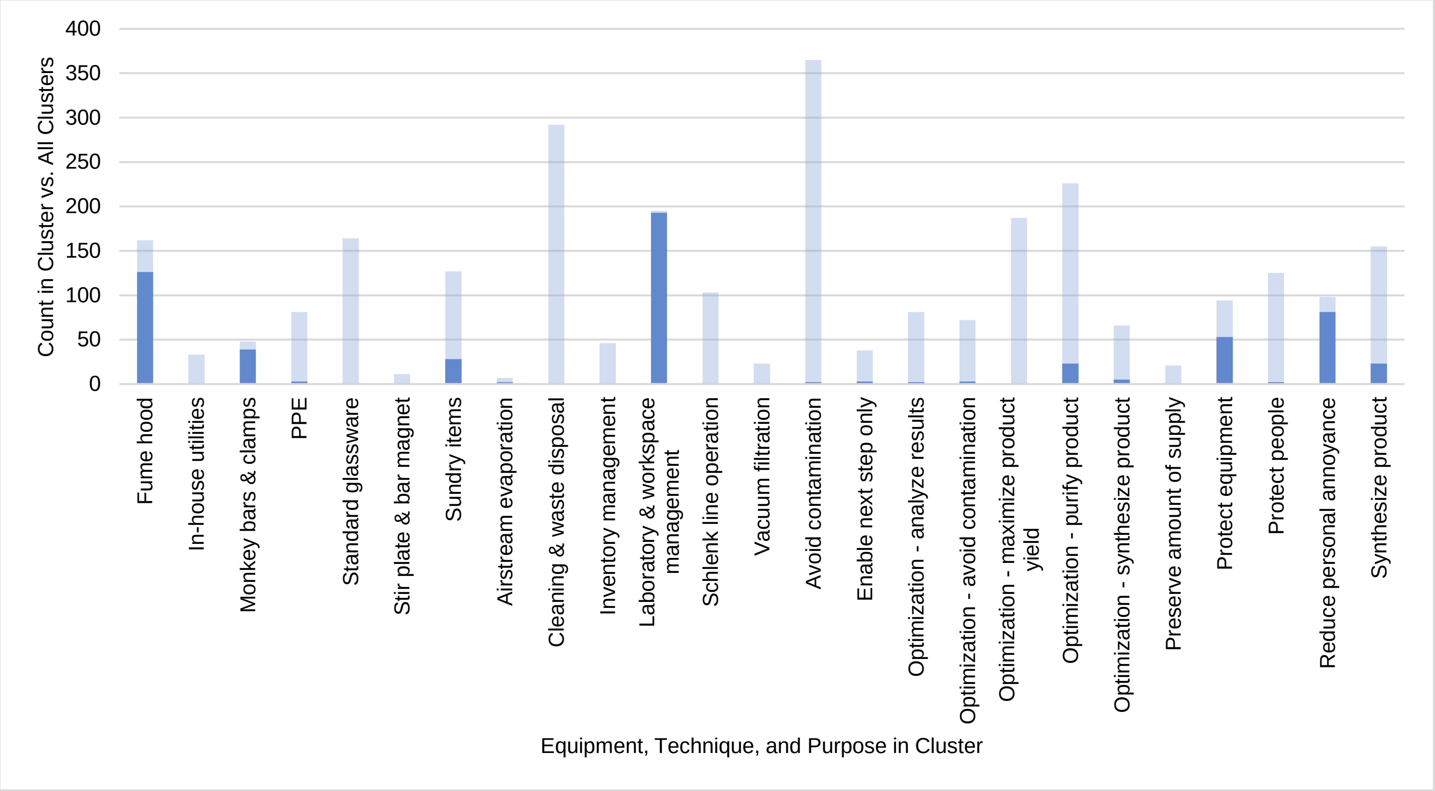


**Figure S9.  Summary of Cluster 6: Staying Safe and Using a Notebook.**

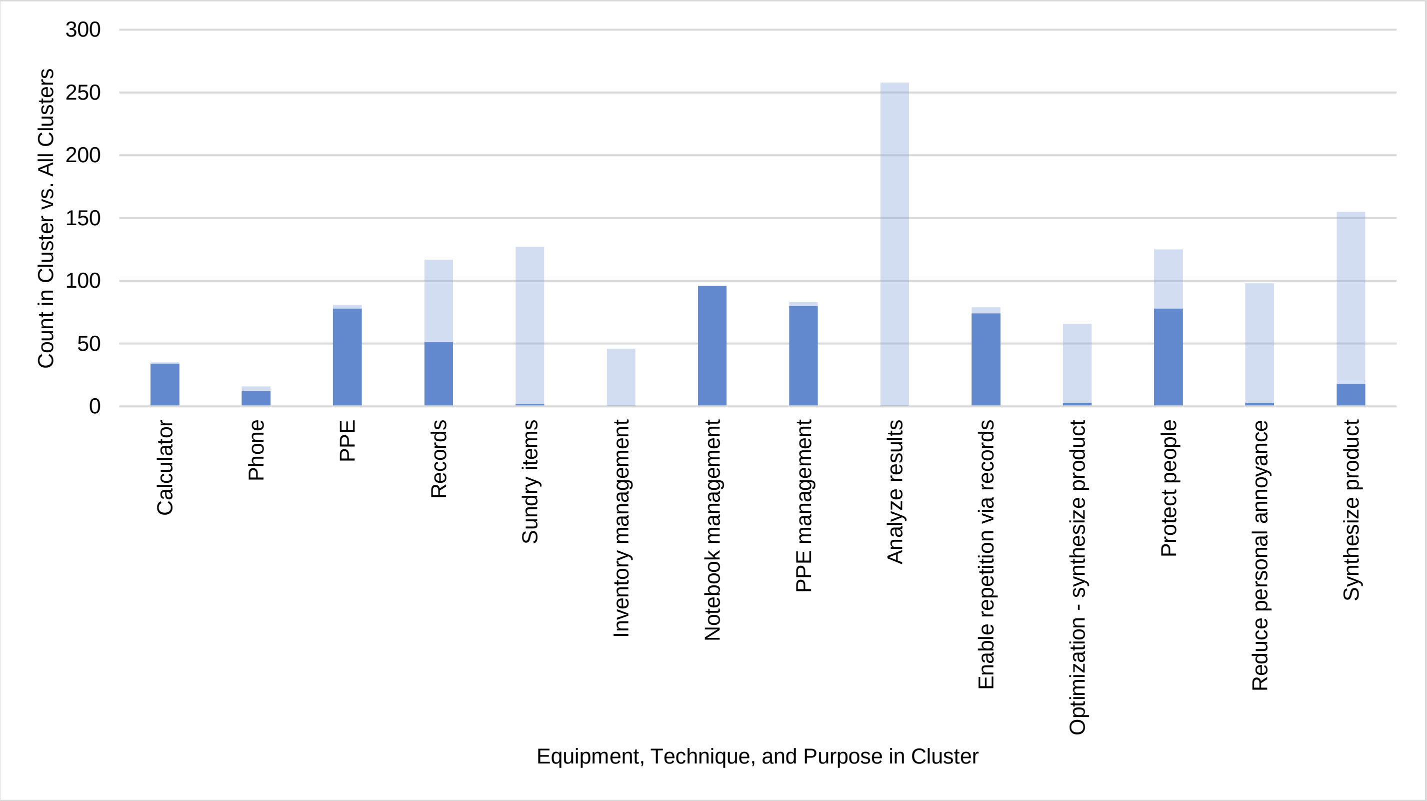

**Figure S10. Summary of Cluster 7: Setting up and Monitoring Reactions.**

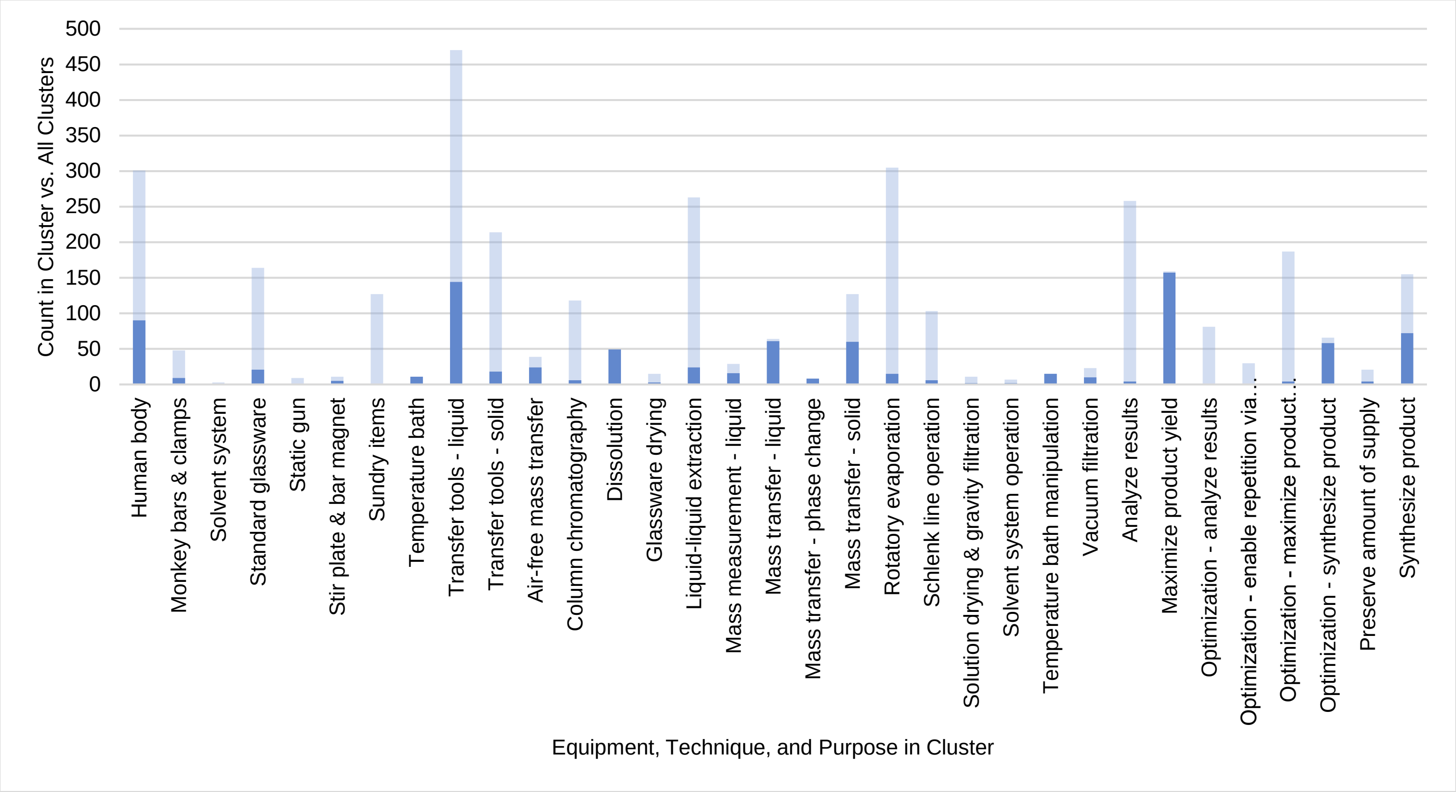


**Figure S11. Summary of Cluster 8: Rotatory Evaporating.**

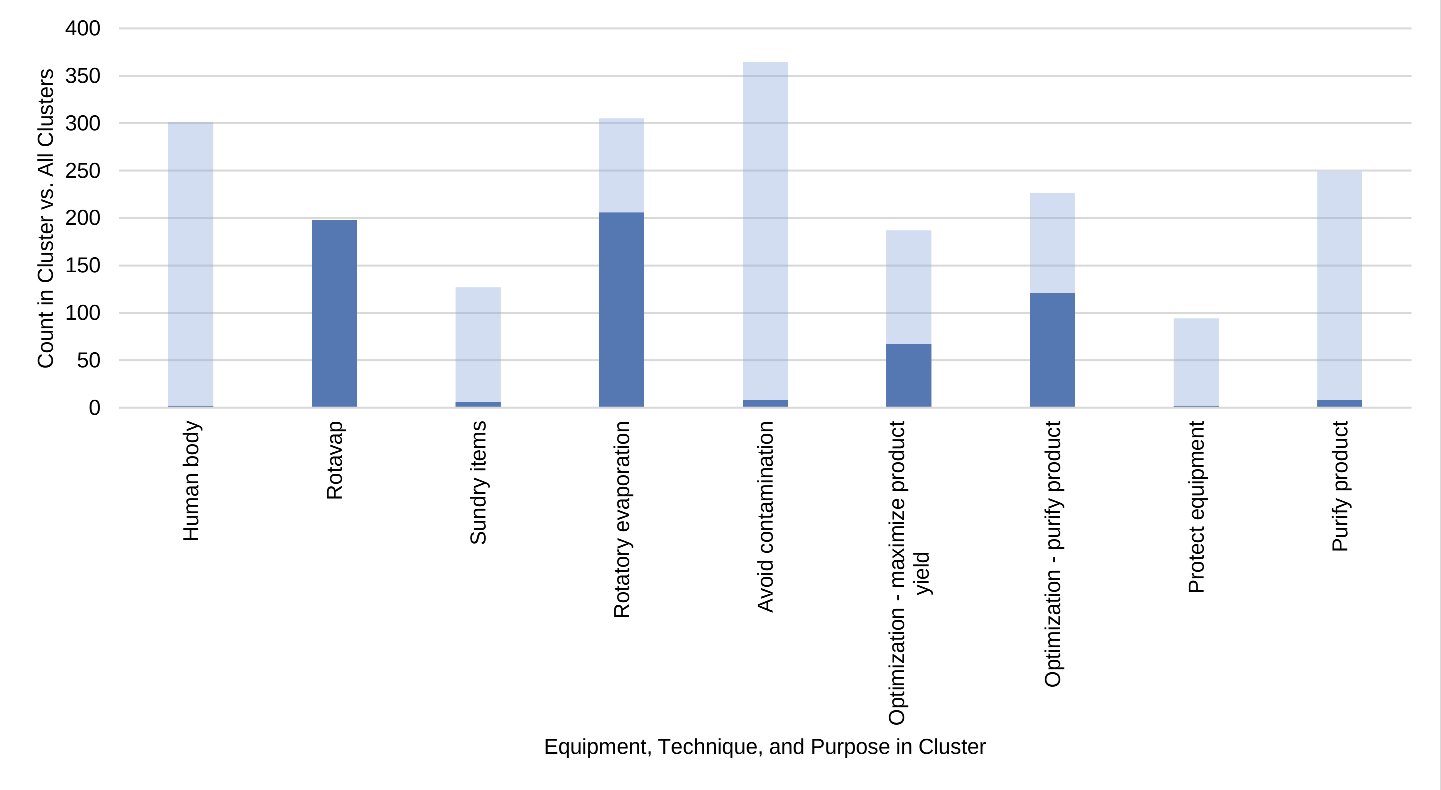

Supplement: Supplementary file 3 — ed2c00809_si_003.pdf [file ed2c00809_si_003.pdf]
